# Determining Physical Mechanisms of Gene Expression Regulation from Single Cell Gene Expression Data

**DOI:** 10.1371/journal.pcbi.1005072

**Published:** 2016-08-23

**Authors:** Daphne Ezer, Victoria Moignard, Berthold Göttgens, Boris Adryan

**Affiliations:** 1 Department of Genetics, Systems Biology Centre, University of Cambridge, Cambridge, United Kingdom; 2 Sainsbury Laboratory, University of Cambridge, Cambridge, United Kingdom; 3 Department of Haematology, Wellcome Trust and MRC Cambridge Stem Cell Institute and Cambridge Institute for Medical Research, University of Cambridge, Cambridge, United Kingdom; Ottawa University, CANADA

## Abstract

Many genes are expressed in bursts, which can contribute to cell-to-cell heterogeneity. It is now possible to measure this heterogeneity with high throughput single cell gene expression assays (single cell qPCR and RNA-seq). These experimental approaches generate gene expression distributions which can be used to estimate the kinetic parameters of gene expression bursting, namely the rate that genes turn on, the rate that genes turn off, and the rate of transcription. We construct a complete pipeline for the analysis of single cell qPCR data that uses the mathematics behind bursty expression to develop more accurate and robust algorithms for analyzing the origin of heterogeneity in experimental samples, specifically an algorithm for clustering cells by their bursting behavior (Simulated Annealing for Bursty Expression Clustering, SABEC) and a statistical tool for comparing the kinetic parameters of bursty expression across populations of cells (Estimation of Parameter changes in Kinetics, EPiK). We applied these methods to hematopoiesis, including a new single cell dataset in which transcription factors (TFs) involved in the earliest branchpoint of blood differentiation were individually up- and down-regulated. We could identify two unique sub-populations within a seemingly homogenous group of hematopoietic stem cells. In addition, we could predict regulatory mechanisms controlling the expression levels of eighteen key hematopoietic transcription factors throughout differentiation. Detailed information about gene regulatory mechanisms can therefore be obtained simply from high throughput single cell gene expression data, which should be widely applicable given the rapid expansion of single cell genomics.

## Author Summary

Many genes are expressed in bursts, which can contribute to cell-to-cell variability. We construct a pipeline for analyzing single cell gene expression data that uses the mathematics behind bursty expression. This pipeline includes one algorithm for clustering cells (Simulated Annealing for Bursty Expression Clustering, SABEC) and a statistical tool for comparing the kinetic parameters of bursty expression across populations of cells (Estimation of Parameter changes in Kinetics, EPiK). We applied these methods to blood development, including a new single cell dataset in which TFs involved in the earliest branchpoint of blood differentiation were individually up- and down-regulated.

## Introduction

Many genes are expressed in stochastic bursts: there are time periods where many transcripts are quickly produced, interspersed randomly with gaps of little or no transcriptional activity. Bursting gene expression was initially proposed as a mechanism to explain why cells in a seemingly uniform cell culture responded heterogeneously to steroids [1]. Two decades later, new live imaging technologies enabled researchers to *observe* transcriptional and translational bursting in real-time, finally confirming that bursting gene expression is a widespread phenomenon [2–4]. In fact, Dar et al. [5] tested 8,000 human genes and found that all of them were expressed in episodic bursts.

**Fig 1:**
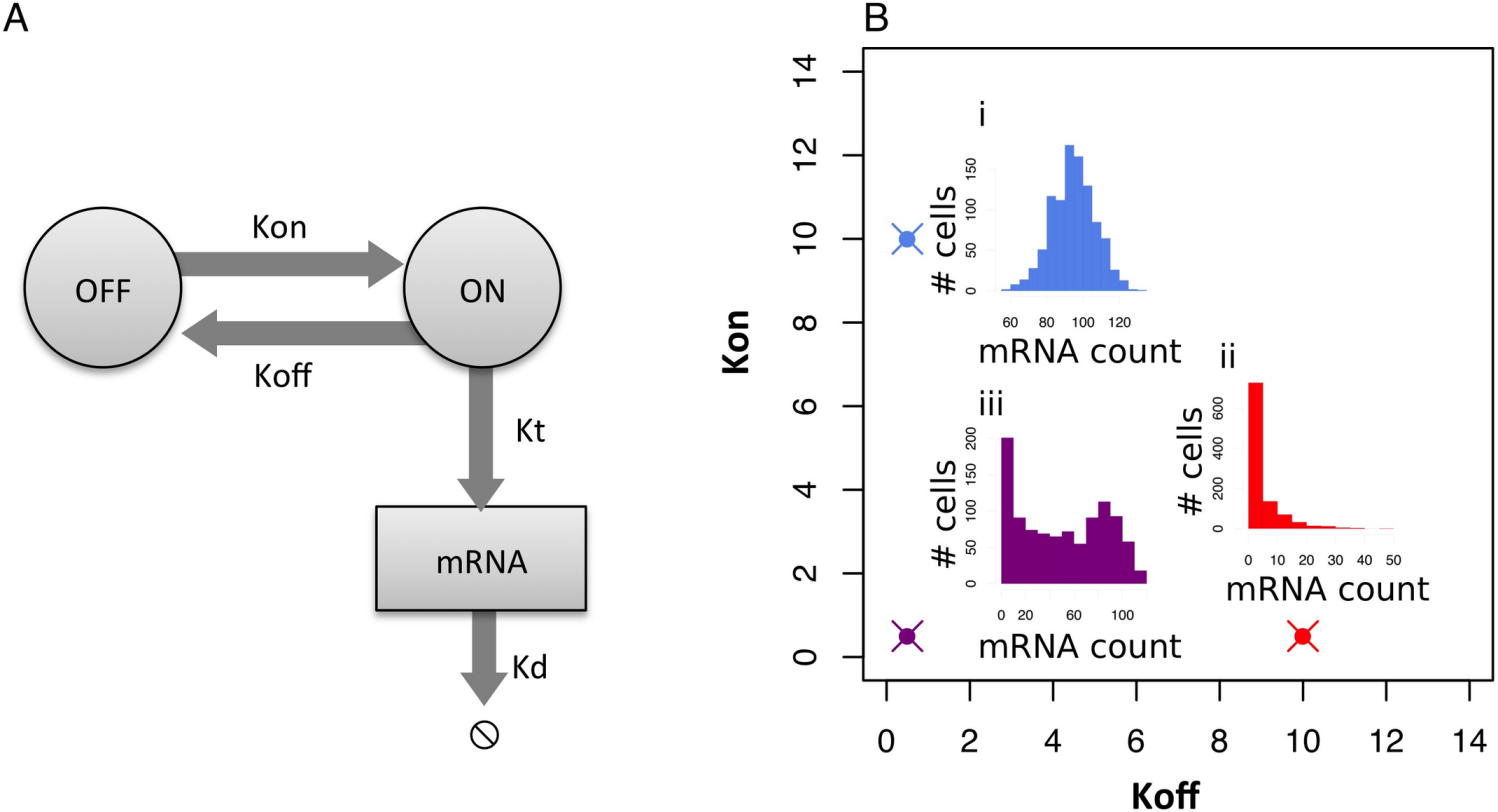
Two-state model of bursty gene expression. The distribution of gene expression in a population of cells is partially caused by bursts of gene expression. (A) In the two state model of bursting gene expression, a gene stochastically turns on at rate *K*_*on*_ and off at rate *K*_*off*_. When the gene is on, it transcribes mRNA at a rate of *K*_*t*_. Note that all three rates are normalised to the rate of mRNA degradation (*K*_*d*_). (B) These kinetic parameters control the shape of the gene expression distribution. In this figure, we keep *K*_*t*_ = 100 and vary *K*_*on*_ and *K*_*off*_. At high *K*_*on*_ the distribution of mRNA transcripts is similar to a Poisson distribution (i), at high *K*_*off*_ the distribution of mRNA transcripts is similar to a negative binomial (ii), and at low values for *K*_*on*_ and *K*_*off*_ the distribution is bimodal (iii).

Ko et al. [6] described bursting gene expression using a *two-state model of gene expression*, depicted in Fig 1A. In this model, each gene can either be in an *on* or an *off* state, and the gene stochastically transitions between these states, with transcription only taking place when the gene is on. The distribution of mRNA across a population of cells is determined by the following three kinetic parameters: the rate the gene turns on (*K*_*on*_), the rate the gene turns off (*K*_*off*_) and the rate of transcription when the gene is on (*K*_*t*_), all normalized to the rate of mRNA degradation [7]. The values of these three kinetic parameters determine the distribution of mRNA transcripts within a population of cells (Fig 1B).

Predicting the rate at which genes turn on, turn off, and transcribe mRNA can provide insights into how genes are regulated. For instance, TFs which are *necessary* for transcription would control the rate at which the genes turn on (*K*_*on*_). One example of such a TF would be a *pioneering factor*, which opens up the chromatin to allow transcription. Brown et al. [8] found that switching between active/inactive states corresponded to distinct chromatin changes in PHO5. This is consistent with the results in Dadiani et al. [9], which show that manipulating nucleosome-disfavoring sequences in yeast can influence the burst frequency. On the other hand, TFs that control *K*_*t*_ are responsible for modulating the levels of gene expression of genes that are already on [10]. For instance, they may be involved in polymerase II (PolII) recruitment or transcriptional elongation. Therefore, estimating these kinetic parameters could help generate hypotheses for gene regulation mechanisms.

Until now, the study of transcriptional bursting has been limited by the available experimental approaches. The most common high-throughput strategies (conventional RNA-seq or qPCR) for measuring gene expression require biological material from thousands of cells. These bulk strategies only measure the *average* levels of gene expression in populations of cells, data that cannot be used to make functional predictions about the bursting dynamics of transcription. While transcriptional bursting can be visualized in real-time in single cells, this is a low-throughput approach which can only measure expression for a single gene per cell [3, 4]. Recently, there has been an emergence of single cell resolution RNA-seq and qPCR technologies, which can observe the full profile of gene expression in a population of cells. However, these are *snapshot* methods, which can only measure gene expression at a single point in time, because they involve lysing the cells.

Nevertheless, preliminary studies have shown that it is possible to utilize the shape of the distribution of gene expression at a single point in time to estimate the kinetic parameters of the two-state model. Raj et al. [11] used a variation of the mathematical analysis done by Peccoud and Ycart [7] to estimate the kinetic parameters in fluorescent in situ hybridization (FISH)-based expression studies, and Kim and Marioni [12] developed a strategy to estimate kinetic parameters in RNA-seq data. In addition, Teles et al. [13] applied a similar approach to a single cell qPCR dataset in the context of a hematopoietic developmental system.

As it is now accepted that bursting dynamics can in principle be resolved from single cell *snapshot* datasets, we can take advantage of this type of analysis to develop new algorithms that can answer a wide range of biological questions. In this paper, we apply our understanding of bursting gene expression to develop a more effective clustering algorithm for single cell gene expression data, which we call Simulated Annealing for Bursty Expression Clustering (SABEC). This can help researchers identify previously uncharacterized sub-populations of cells within their single cell data. Secondly, we develop a statistical tool for identifying whether the burst frequency (*K*_*on*_ or *K*_*off*_) or burst magnitude (*K*_*t*_) are the source of gene expression variations between experimental samples. Instead of simply observing whether gene expression increases or decreases across two populations of cells, this Estimation of Parameter changes in Kinetics (EPiK) toolkit allows researchers to make inferences about the underlying regulatory mechanisms affecting gene expression. We provide a complete pipeline in R for analyzing single cell qPCR data, including data normalization steps, SABEC clustering and EPiK cluster comparisons.

This pipeline can be utilized in a wide range of biological contexts. We apply it to study how some of the key transcription factors in hematopoiesis are being regulated, discovering that the hematopoietic stem cells studied in Moignard et al. [14] formed two distinct sub-populations, distinguishable by Tel and Gata1 expression levels. Then, by manipulating the expression levels of two of the TFs involved in early differentiation (Gfi1 and Gata2), we predict that the primary mechanisms by which these TFs influence their downstream targets is by manipulating *K*_*on*_. Finally, we compare the mechanisms by which cell surface markers are regulated in healthy and leukemic cells based on data previously reported in Guo et al. [15].

## Results

### A pipeline for analyzing single cell gene expression data

**Fig 2:**
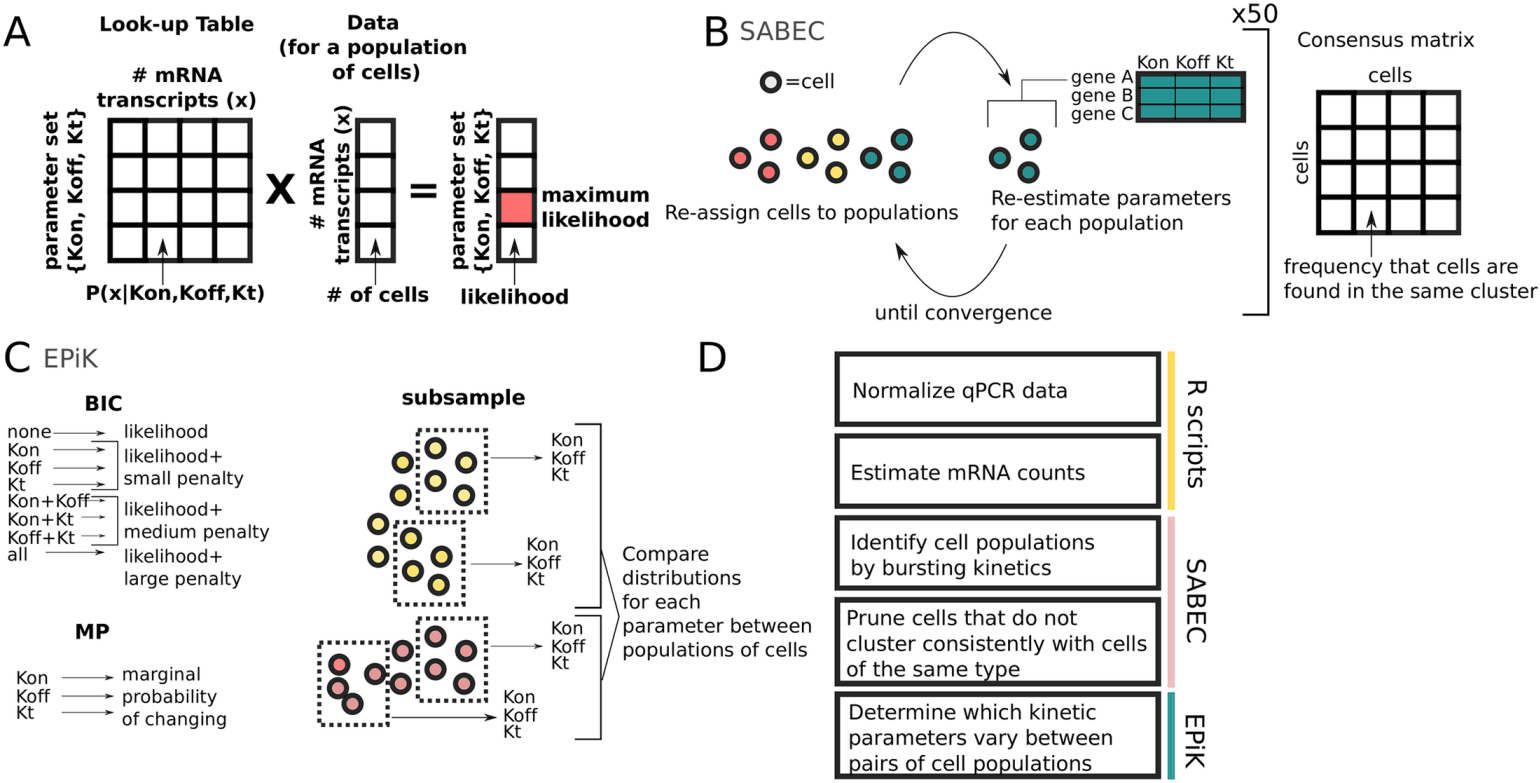
Pipeline for the analysis of single cell gene expression data. This paper develops several new tools for single cell analysis. (A) The kinetic parameters are estimated using a large look-up table. The likelihoods for each possible parameter set are found by multiplying the experimental data by the lookup table, and then the maximum likelihood is identified. (B) The SABEC algorithm is used to identify clusters of cells with uniform bursting kinetics– this iterative algorithm alternates between assigning cells to clusters and estimating bursting kinetic parameters for each cluster. This clustering algorithm is run 50 times and the results are summarized in a consensus matrix, which represents the frequency of each pair of cells being found in the same cluster. (C) Finally, the EPiK tool identifies the most likely set of parameters to have varied for a gene across two different cell populations, using a combination of the Bayesian Information Criterion (BIC), Marginal Probability (MP), and a Subsampling-based method, as described in (C). BIC calculates the likelihood for each possible set of parameters, penalised by the number of free parameters. The MP score calculates the likelihood of each parameter changing, independent of the behaviour of the other parameters. In the subsampling method, random sets of cells are selected, the kinetic parameters estimated for each population of cells; then, the distributions of estimated kinetic parameters are compared. (D) These new tools are combined to form a pipeline for analyzing single cell qPCR data. The details for each step of this pipeline are described in the *Methods*.

We have developed a pipeline for analyzing single cell qPCR data in order to determine the mechanism by which TFs were regulated during differentiation (Fig 2).

The input data consists of single cell qPCR data for a set of genes, where the cells have been sorted into their *expected* cell populations, using fluorescence-activated cell sorting (FACS). There are two main outputs of the pipeline. Firstly, cells are divided into populations (clusters) based on their bursting behaviour– each gene has the similar rates of activation (*K*_*on*_), rates of inactivation (*K*_*off*_) and rates of transcription (*K*_*t*_) across all cells within each cluster. Secondly, the pipeline compares between each pair of cell populations and predicts the mechanism by which each gene changes its expression– by changing the rate the gene turned on and off or by changing the rate of transcription once the gene is already active.

A key component in the pipeline is our strategy for estimating the kinetic parameters. Previous approaches for estimating the kinetic parameters were slow to compute, because they involved iterative algorithms, such as simulated annealing [13], expectation maximisation [11], or Gibbs Sampling [12]. Meanwhile, we have a pre-computed look-up table for *P*(*x*|*K*_*on*_, *K*_*off*_, *K*_*t*_), the probability of seeing *x* mRNA transcripts, given a set of kinetic parameters (Fig 2A and Eq 1). This table can be used to compute the most likely set of kinetic parameters using a single matrix multiplication– a very efficient computation (Eq 2).

In the pipeline, first we normalize the measurements from the qPCR and transform them into estimated mRNA counts (See Eq 6). Next, these measurements are input into a newly developed unsupervised clustering algorithm (SABEC) which identifies populations of cells with uniform bursting kinetics. SABEC is an iterative algorithm that starts by randomly assigning cells to clusters; next, the algorithm alternates between estimating the kinetic parameters for each gene within each cluster (using Eq 2) and probabilistically re-assigning cells to clusters based on their likelihood of belonging to each cluster (using Eq 9), until the algorithm converges– i.e. when fewer than 5% of cells swap clusters. SABEC is run fifty times using different initial sets of randomly assigned clusters, and the results are summarised as a consensus matrix– a matrix that records the number of times two cells were grouped together. The number of clusters can be determined by one of the following methods: the proportion ambiguously clustered (PAC), Variable Information (VI) or the corrected Rand score (See Fig 2B).

Finally, each *pair* of populations is compared using a statistical tool we developed called EPiK, in order to identify whether each gene is regulated by modifying the rate the gene turns on (burst frequency) or the level of transcription once the gene is active (burst magnitude). EPiK is a compilation of three different methods: the Bayesian Information Criteria (BIC), a Marginal Probability (MP) Score, and a subsampling method. Since for each gene there can be between 0 and 3 parameters that can differ across a pair of populations, BIC identifies the set of parameters most likely to differ after penalising parameter sets that are larger (See Eq 12). The MP score separately calculates the likelihood of each parameter changing, independent of whether the other parameters change or stay the same (See Eq 15). In the subsampling method, the kinetic parameters are calculated for small random subsets of cells, then the distributions of estimated kinetic parameters for the subsets are compared by the Kolmagorov-Smirnov statistic(Fig 2C).

All together, our R data processing scripts, SABEC, and EPiK come together to form a pipeline that utilizes the mathematics of bursting gene expression to determine how genes are differentially regulated across populations of cells (Fig 2D).

### Validation of methods with *in silico* datasets

These methods were all tested *in silico* using data sets that have similar structures to the experimental datasets. Each of the three methods introduced in this paper (ML approach for kinetic parameter estimation, SABEC and EPiK) required a different set of simulated data.

The ML method for kinetic parameter estimation was benchmarked populations of cells with known kinetic parameters to allow us to quantify the accuracy of the method. 3,000 parameter sets were randomly selected (uniformly distributed) with *K*_*on*_ between 0 and 5, *K*_*off*_ between 0 and 20, and *K*_*t*_ between 0 and 600. We randomly sampled 10% of the transcripts from each of the simulated cell, to represent the technical noise caused by the loss of 90% of the starting material during sample preparation. We included a stochastic loss of mRNA transcripts to account for material loss during cDNA library construction– Islam et al. [33] estimates that only 48% of transcripts are reverse transcribed into cDNA, and Wu et al. [34] could capture 42% of the total unique transcripts that were identified in bulk RNA-seq.

The simulated datasets for testing the SABEC method were chosen to be as similar to the experimental datasets as possible. 100 simulation sets were generated, with each completely parallel to the Moignard dataset; Each simulation set consisted of five populations of 124 cells with 18 genes each, with their kinetic parameters equal to those estimated by the ML method for the experimental data.

Finally, to test the final method of comparing kinetic parameters between two populations of cells, 1600 simulated datasets were generated, each one consisted of a pair of populations of 124 cells with 1 gene each. There are eight combinations of kinetic parameters that can change (none, all, three ways one parameter can change and three ways two parameters can change). Two hundred pairs of populations were selected for each of these eight scenarios, with 100 simulations with 0 < *K*_*off*_ < 5 and 100 simulations with 5 < *K*_*off*_ < 10. The range of the other parameters were 0 < *K*_*on*_ < 5 and 0 < *K*_*t*_ < 600. In these simulations we also simulated the random loss of 90% of the mRNAs.

**Fig 3:**
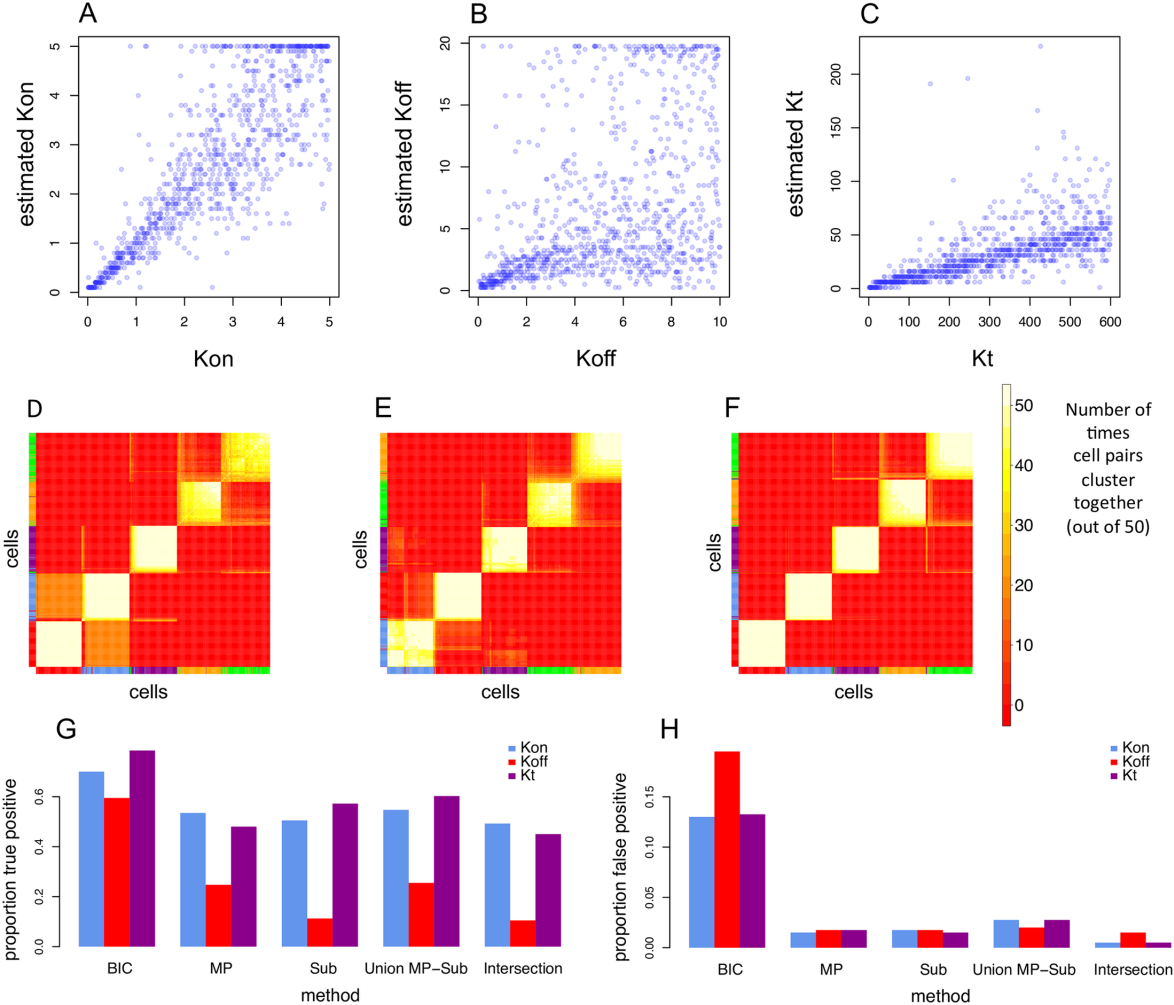
*In silico* validation of single cell analysis pipeline. In order to estimate the accuracy of the new tools developed in this paper, each tool was evaluated against simulated datasets. (A-C) The kinetic parameter estimation method was tested against simulated data in which 90% of the data was randomly discarded, to simulate the loss of biological material through inefficient cDNA preparation. The known kinetic parameters and the estimated kinetic parameters were compared for *K*_*on*_ (A), *K*_*off*_ (B) and *K*_*t*_ (C). (D-F) Next, SABEC was tested against simulated datasets that had the same kinetic parameters as those estimated for the cell populations in the Moignard single cell dataset (124 simulated cells for each of the 5 cell populations)—SABEC was tested on 100 of such simulated datasets and the robustness of the clustering was measured by calculating the proportion of ambiguously clustered cells (PAC). This figure depicts a hierarchical clustering of the consensus matrices that come from (D) the dataset whose clustering had the worst PAC score, (E) a randomly selected dataset, and (F) the dataset whose clustering had the best PAC score. The coloured bars along the side and bottom represent the true class labels of each cell. (G-H) Next, the true positive and false positive rates were calculated for each proposed component of EPiK, the union of the MP and subset method, and the intersection of all three methods. For the MP and Subset methods, thresholds were set so the false positive rate would be approximately 2%– receiver operating characteristic (ROC) curves for these are found in S13 and S14.

Even after simulating 90% loss of biological material, the predicted parameters correlated well with their real values, although *K*_*off*_ was the most difficult parameter to predict (See Fig 3A–3C), especially at wider parameter ranges (S1 and S2 Figs), but it still performed better than the Kim and Marioni Method (S3 Fig). These *in silico* results emphasize that the cDNA library efficiency can have a large impact on the absolute values of predicted parameters, which suggests that raw parameter values are not comparable across different experiments. SABEC also accurately predicted clusters of simulated datasets, with similar population structures and parameter ranges to the experimental dataset (Fig 3D–3F). Finally, we found that the EPiK method was very conservative, with approximately a 0.05% false positive rate when the methods were intersected (Fig 3G and 3H). Further details about the validations and comparisons with alternative methods are described in depth in the *Methods*. Next, we applied these methods to experimental datasets.

### Single cell analysis of hematopoietic stem cells and progenitors

The dataset we use to test our pipeline comes from Moignard et al. [14], which is a high quality single cell qPCR dataset that includes approximately 124 cells each from five different populations of cells during hematopoeisis. Hematopoiesis is the process by which hematopoietic stem cells (HSC) in the bone marrow differentiate into different types of red and white blood cell types. This process can be depicted as a differentiation tree, in which each cell must make multiple “decisions” at each branching point in the tree that will determine its final cell fate. Moignard et al. [14] includes two key branching points: i) HSC cells can become lymphoid-primed multipotential progenitors (LMPPs) or premegakaryocytes (PreMegEs, also referred to as PreMs in figures) and ii) LMPP cells can become granulocyte-macrophage progenitors (GMPs) or common lymphoid progenitors (CLP).

The focus of this study is on the densely interconnected TF regulatory network that has been shown to contribute to cell fate decisions. There is strong evidence that at least seven of these TFs directly interact with one another, potentially forming a SCL/Lyl1/Gata2/Runx1/Lmo2/Fli-1/Erg heptad, with some TFs directly binding to the DNA (such as Gata2 and SCL) and other TFs serving a bridge between the DNA bound components (such as LMO2) [16]. Other key TFs that were profiled in Moignard et al. [14] were PU.1, Meis1, Hhex, Tel, Nfe2, Eto2, Mitf and Ldb1.

There are a number of open questions about the differentiation of HSC into the various progenitor cell populations. Firstly, although the cells were assigned to their populations via FACS, it is unclear whether these sub-populations are truly uniform. For instance, some HSC cells may be biased towards self-renewal or producing cells in the lymphoid or myeloid lineages.

Secondly, the specific mechanisms by which these TFs are being regulated are as yet uncharacterized. Influencing the rate at which genes turns on (*K*_*on*_) could increase the proportion of cells with an active copy of a gene; whereas manipulating the transcription rate (*K*_*t*_) would result in there being higher concentrations of mRNA, while maintaining the same proportion of cells with an active gene. In hematopoiesis, cellular TF concentrations help determine which branch the cell will take as it differentiates towards its final cell fate. Therefore, choosing to manipulate *K*_*on*_ instead of *K*_*t*_ could influence the proportion of cells that enter a certain differentiation trajectory. In addition, since the expression of these TFs is tightly linked, one gene’s bursting dynamics could have repercussions on the dynamics of the entire network.

### Hematopoietic stem cell populations have heterogeneous bursting kinetics

Although the cells that we study have been sorted into distinct subpopulations using FACS, this does not guarantee that the populations are indeed transcriptionally uniform. Some cells may be misclassified by FACS (expected misclassification rate is 1% for the Moignard dataset), and some of the known populations may be composed of as yet unidentified subpopulations. In addition, extrinsic variability, such as having cells in different stages of the cell cycle, could cause the populations to be heterogeneous.

It can be difficult to accurately identify homogenous subpopulations using standard clustering approaches like K-means or hierarchical clustering (S5 Fig). For instance, K-means is most effective when each cluster is normally distributed and has similar variances. Due to bursting, gene expression is unlikely to come from such a distribution; gene expression distributions can look like a Poisson distribution, a negative binomial distribution, or even a bimodal distribution, depending on *K*_*on*_, *K*_*off*_, and *K*_*t*_. Therefore, we developed a simulated annealing strategy (Simulated Annealing for Bursty Expression Clustering, SABEC), which takes into account bursting gene expression, in order to have more robust clustering. SABEC was rigorously tested against simulated datasets that were designed to be as close as possible to the structure of the experimental data (S6 Fig). Additionally, the algorithm was tested on a wide range of simulated datasets, to see if this method could perform well under varied conditions, such as on data with different numbers of genes measured in each cell and different numbers of cells in each population (S7 Fig).

**Fig 4:**
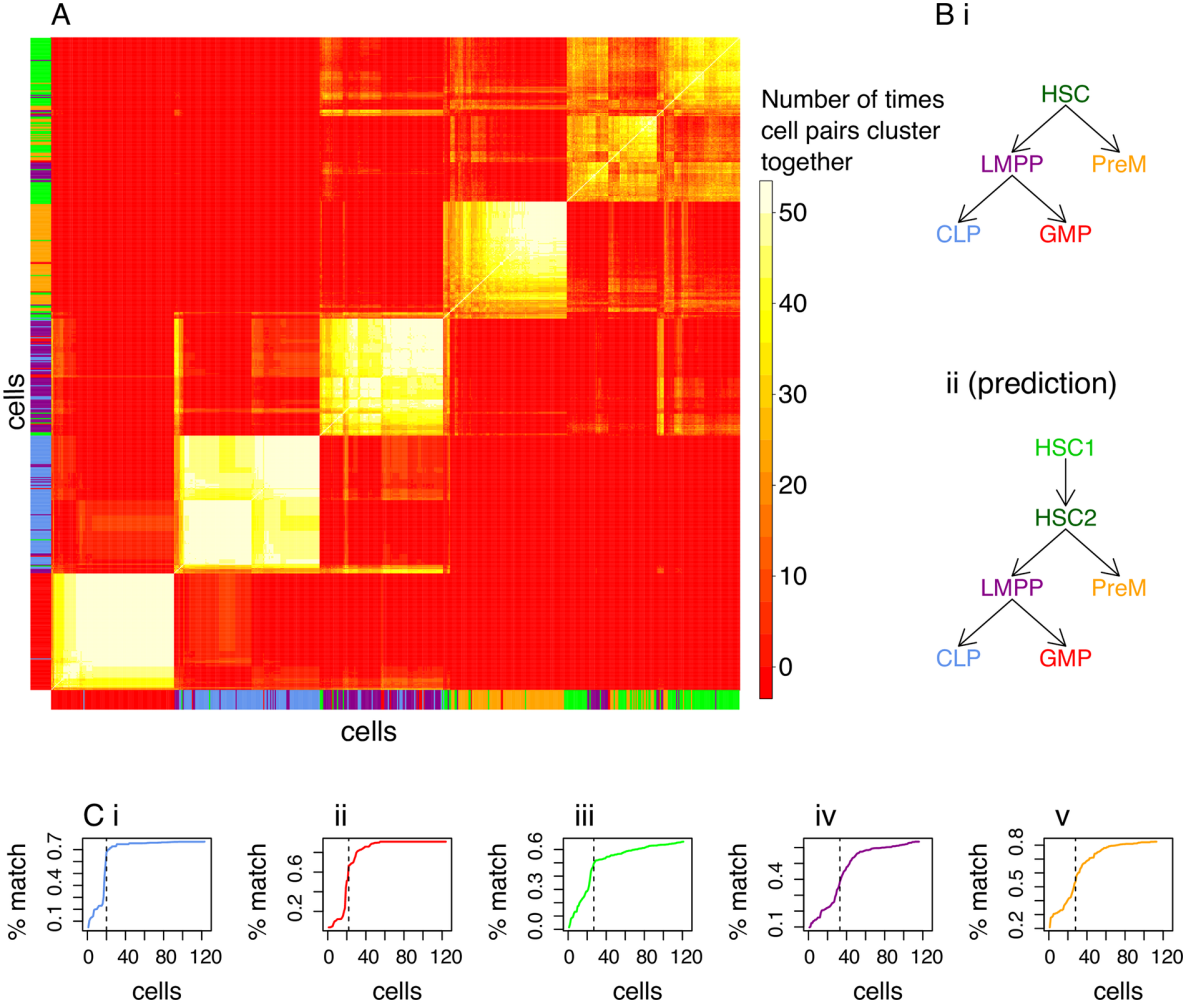
Application of SABEC to hematopoietic stem cells and progenitor populations. SABEC was used to identify subpopulations of cells in the Moignard et al. dataset and to identify specific cells that do not cluster well with the other cells of the same type. (A) Hierarchical clustering was applied to the SABEC consensus matrix to reveal six subpopulations of HSC in subfigure. (Bi) The expected hematopoiesis differentiation tree is shown–the names of the cell populations are color-coded to match the vertical and horizontal bars in A. (Bii) We hypothesized a possible alternative differentiation tree, based on the clustering results. (C) Another application of this clustering method is to remove possible outliers, which may have arisen due to poorly sorted cells or extrinsic variability. Within each cell population (i.e. CLP (i), GMP (ii), HSC (iii), LMPP (iv) and PreM (v)), we sorted the cells by how often the cells are clustered with other cells from the same FACS label (% match). The vertical lines depict thresholds selected to approximately correspond to the region with the steepest slope– cells to the left are disregarded as outliers in the EPiK analysis.

Next, SABEC was applied to the experimental single cell qPCR data from Moignard et al. [14]. The final clustering is depicted in Fig 4A. Although the predicted hematopoiesis differentiation tree is expected to look like Fig 4Bi, we found that HSC is divided into two distinct subpopulations. One of these populations had 12.8% of cells expressing Gata1, while the other population had none. Since Gata1 is uncommon in HSC cells, but often found in multi-potent progenitor populations (MPP, the earliest progenitor population formed by HSCs [17]), we hypothesized that the populations of cells without Gata1 may be self-renewal HSCs and the other may be HSCs poised for differentiation.

Our clustering with SABEC was based solely on the expression of 18 TFs. However, Moignard et al. [14] also profiled cell surface protein tyrosine kinase c-Kit. It is known that low levels of c-Kit correlate to greater self-renewal potential in HSCs [18]. Even though all of the HSC cells were positive for the c-Kit protein according to FACS, not all the cells had high levels of c-Kit transcripts. Our hypothesized self-renewal HSC population had significantly lower levels of c-Kit than our poised-to-divide population (p-value 9.117e-06 with Kolmogorov-Smirnov test). Even though c-Kit was not one of the genes used to cluster the cells, there was substantial difference in expression levels, suggesting the tree topology depicted in Fig 4Bii. Further evidence for this arrangement is in S9 Fig.

The SABEC algorithm has provided us with subpopulations that appear to have uniform bursting kinetics. In the next section we will identify which kinetic parameters were most likely adjusted across each pair of cell populations, using EPiK. EPiK works best when cell populations have uniform bursting dynamics; S11 Fig shows how having mixed cell types can influence estimates of kinetic parameters. Therefore, we remove cells that do not cluster well with other cells of the same type, with cutoff thresholds designated by the vertical lines in Fig 4C, which is determined by the region of the curve with the steepest slope. However, this pruning protocol could bias parameter estimation if it were to eliminate cells that are falsely identified as outliers (S10 Fig). Therefore, we run EPiK both on the pruned and unpruned datasets, and only consider parameters that are consistently found to have changed under both conditions. It is important to note that the proposed outlier cells may be of biological interest– for instance, they may be rare cell types or cells in the process of differentiation. The pruned dataset is only for use to boost the accuracy of predictions with EPiK, but *all* cells should be observed for other types of analysis.

### Transcription factors involved in hematopoiesis are regulated by different mechanisms at each stage of differentiation

EPiK incorporates three different metrics for evaluating whether kinetic parameter changes are significant. By taking the intersection of these three prediction methods, the false positive rate decreases without a significant drop in the true positive rate. This gives us a very conservative list of probable kinetic parameter changes (a 0.05% false positive rate with our simulated dataset).

The first method is the Bayesian Information Criterion (BIC) (S12 Fig), and the second method is the marginal probability (MP), which is the log likelihood of a certain parameter being varied, independent of whether the other two parameters vary or stay the same (S13 Fig). In the third method, we repeatedly subsample cells from each population and estimate the kinetic parameters for each subset, comparing the maximum distance between the cumulative density functions of the distributions (S14 Fig).

These three methods were applied to the experimental data from Moignard et al. [14]. Each of the methods is based on slightly different assumptions, so they each have different distributions of parameters being varied (See S15 Fig). For instance, BIC predicts that *K*_*off*_ is adjusted in a few cases, but this is not deemed significant by either the MP or subset methods. In addition, there are more significant parameter changes in the case of the pruned populations compared to the complete populations, because these populations are more distinct from one another.

**Fig 5:**
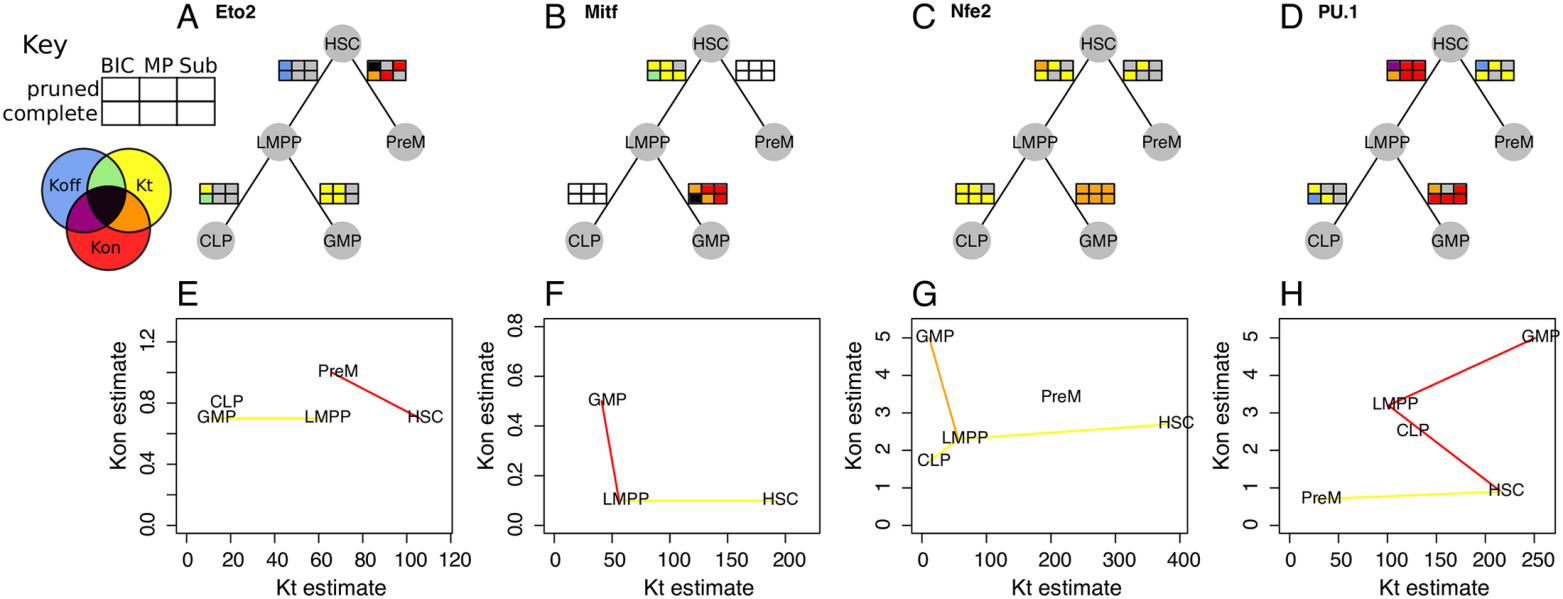
Transcription factor-specific kinetic parameter changes. For each specific gene, it is possible to use EPiK to predict which kinetic parameters vary throughout differentiation. (A-D) For each transition between populations of cells, the kinetic parameter changes are predicted for each TF using all six methods shown in the key, which we represent by six colored rectangles– the color corresponds to the parameters predicted to have changed, as designated in the key. If there are not enough cells with a certain gene expressed in order to calculate parameter changes, then the rectangles are depicted as white. The results are shown for a selection of TFs: Eto2 (A), Mitf (B), Nfe2 (C), and PU.1 (D). (E-H) To demonstrate that EPiK provides reasonable results, we depict the maximum likelihood estimates for *K*_*t*_ and *K*_*on*_ for each population of cells (pruned dataset). If there is a predominant change in *K*_*on*_ (red), *K*_*t*_ (yellow), or both variables (orange), then these populations are connected by lines of the corresponding color.

We can now take a closer look at a few examples of TFs that have predicted kinetic parameter changes during differentiation (Fig 5). The predictions from each method are drawn along the branches of the hematopoiesis differentiation tree (Fig 5A–5E). To demonstrate the magnitude and direction of these kinetic parameter changes, Fig 5E–5H illustrates the kinetic parameter estimates for each population of cells.

In Fig 5A and 5F, four out of the six methods predict that Eto2 is up-regulated by increasing *K*_*on*_ (red) during the HSC to PreM transition, which is consistent with Eto2 having a role in increasing the proportion of cells that differentiate into PreM [19].

One striking feature of Fig 5A–5E is that Eto2, Mitf, and PU.1 are regulated by different kinetic parameters in different stages of blood differentiation. PU.1 has known cell-type-specific enhancer elements [20], and our results may suggest that each of these may regulate PU.1 through different mechanisms. On the other hand, Nfe2 is *consistently* regulated by *K*_*t*_ throughout differentiation.

**Fig 6:**
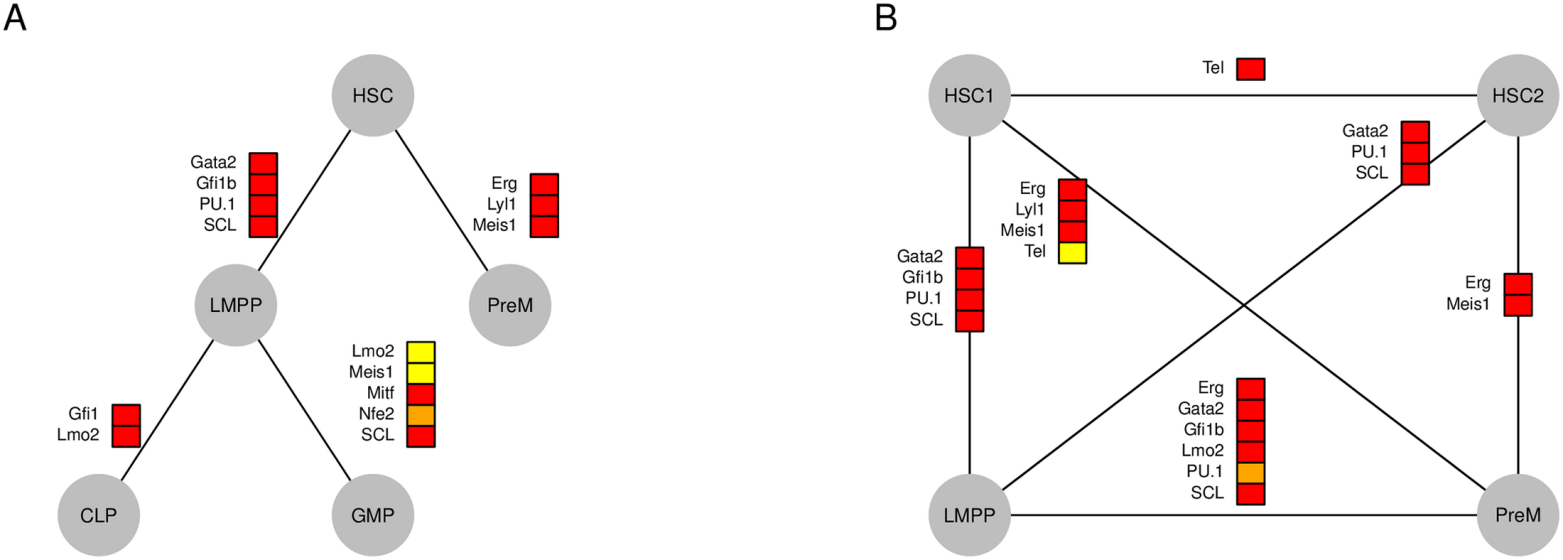
Most significant kinetic parameters variations during hematopoiesis. This figure illustrates kinetic parameter change predictions that are consistent across all six methods illustrated in the key of Fig 5– in other words, this is the intersection of BIC, MP, and the subset methods, as applied to both pruned and un-pruned datasets. Just as in Fig 5, red boxes designate that *K*_*on*_ varies, yellow boxes designate that *K*_*t*_ varies and orange boxes designate that both parameters change. A positive/negative signifies that gene expression increases/decreases along the line (top to bottom; or in the case of horizontal lines left to right). (A) The most significant kinetic parameter changes are shown for the expected hematopoietic tree. (B) Based on the SABEC clustering analysis, it appeared as if there were two distinct HSC subpopulations– the differences between these two subpopulations and LMPP and PreM subpopulations were also calculated.

In some instances, all six methods come to a consensus as to which kinetic parameter was the source of gene expression variability for a particular TF, and these are shown in Fig 6A. Throughout hematopoiesis, most of the differences in gene expression come from changes of *K*_*on*_, but Lmo2, Nfe2 and Meis1 are regulated by *K*_*t*_ in the transition from LMPP to GMP. Recall that in the previous section, we identified that HSC forms two distinct sub-populations. We compared the two HSC sub populations with their child populations (LMPP and PreM) (see Fig 6). Between HSC1 and HSC2 only Tel seemed to consistently change its kinetic parameter (*K*_*on*_). As expected, the cell population with higher Gata1 and c-Kit expression has more in common with LMPP and PreM cells than the other HSC subpopulation.

In summary, our methods have allowed us to not only identify which genes have changed their expression, but also what physical mechanism caused that change.

### Gata2 and Gfi1 may regulate transcription by influencing the rate genes turn on

In the previous section, we identified TFs that were regulated by *K*_*on*_ or *K*_*t*_ during blood differentiation, but it is unclear which TFs were *controlling* these changes. It may be possible to discover the specific mechanistic role of a TF by manipulating its expression experimentally and then calculating the change in kinetic parameters of its downstream targets.

We decided to focus on the Gfi1-Gfi1b-Gata2 subnetwork that was identified as being important at the first branching point of HSCs to LMPP and PreMegEs [14]. Primary HSCs are difficult to isolate in large quantities, culture and manipulate, so we turned to HPC7 cells, a model cell line for hematopoietic stem and progenitor cells which has some differentiation potential towards more mature blood cells [16, 21]. Similarly to HSCs, HPC7 cells express Gata2 and Gfi1b, but little or no Gfi1. We therefore up-regulated Gfi1 expression and down-regulated Gata2 expression and performed single cell gene expression analysis for the gene set described by Moignard et al. [14], as well as some additional genes involved in HSC differentiation. We analysed 81 cells expressing an shRNA against Gata2 and 77 cells control cells expressing an shRNA against Luciferase, and 72 cells overexpressing Gfi1 and 45 control cells expressing an empty vector.

There are a number of strategies by which a TF could reduce gene expression: by decreasing the rate a gene turns on, by increasing the rate a gene turns off, or by decreasing the rate of transcription of an active gene, and each of these strategies would result in different temporal dynamics of gene expression bursting. All six methods came to a consensus that up-regulating Gfi1 seemed to significantly alter Erg, Gfi1b, Hhex and Mpl, by lowering *K*_*on*_. Previous research suggests that Gfi1 is usually a repressor that either keeps the chromatin in a condensed state or actively competes for binding with activators [22]. Both of these mechanisms of action are consistent with Gfi1 down regulating its targets by lowering *K*_*on*_. Based on ChIP-seq experiments from Sanchez-Castillo et al. [23], Gfi1 binds in or near all four of these potential targets (see S1 Table).

In the other experiment, Gata2 was down-regulated; however, this was not a complete knockdown, with only a slight overall decrease in expression (S20 Fig). All six methods suggest that Gfi1b expression was decreased via a change in *K*_*on*_ as Gata2 levels decreased. In the pruned population of cells, Procr (also known as EPCR, a known target of Gata2) had higher *K*_*on*_ after the knockdown [24].

When Gata2 and Gfi1 were down- and up-regulated, our methods could only detect changes in *K*_*on*_. Therefore, we can hypothesize that this could be the mechanism of action of these two TFs.

### Gene expression variations in leukemia

**Fig 7:**
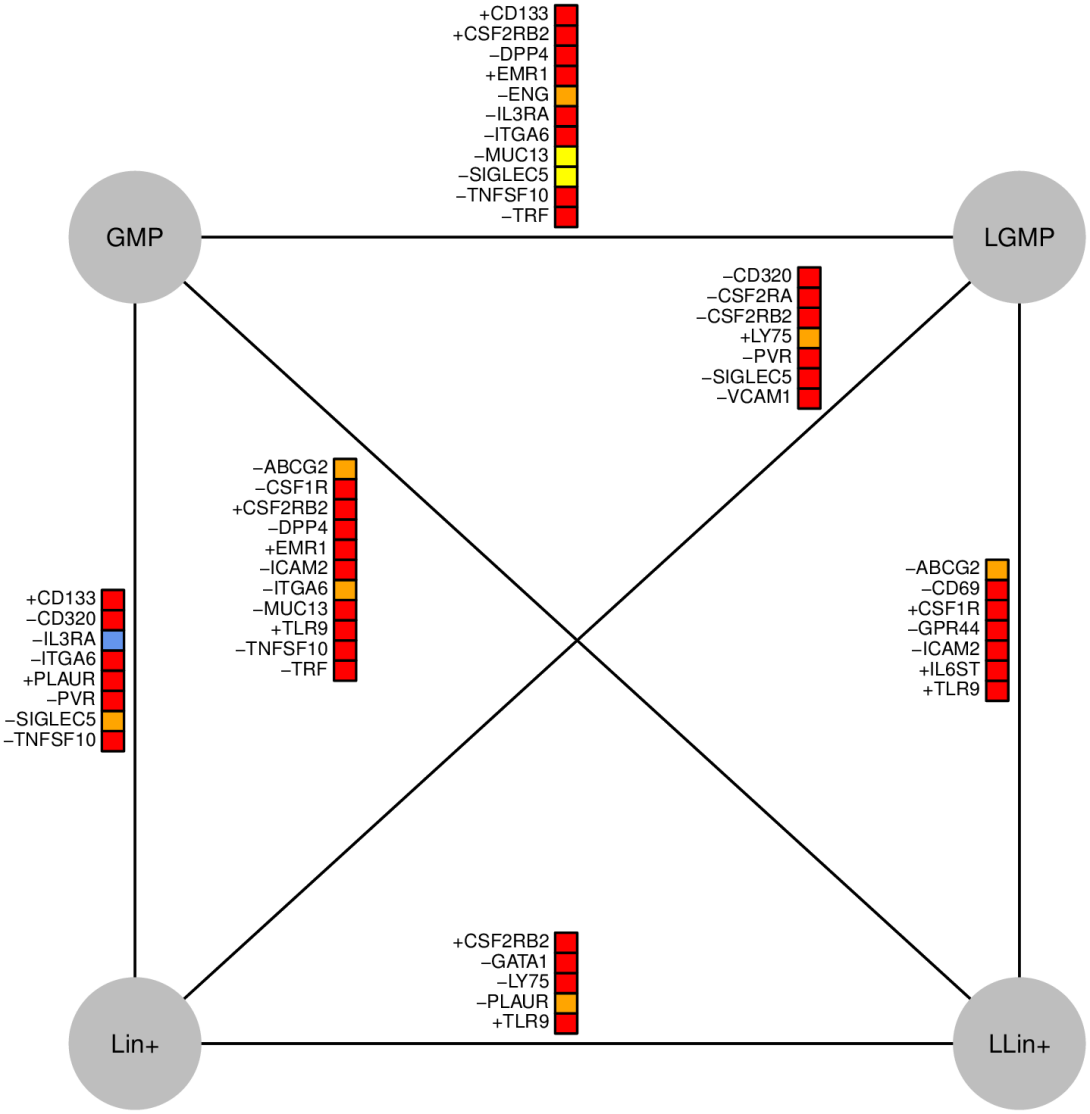
Kinetic parameter differences between leukemic and healthy cells. This figure illustrates kinetic parameter change predictions for the single cell qPCR data in Guo et al. Just as in Figs 5 and 6, red boxes indicate that *K*_*on*_ varies, yellow boxes indicate that *K*_*t*_ varies and orange boxes signify that both parameters change. The blue box indicates that *K*_*off*_ varies. A positive/negative signifies that gene expression increases/decreases along the line (top to bottom; or in the case of horizontal lines left to right).

Our pipeline for the identification of differential kinetic parameter values can also be applied to compare healthy and diseased cell populations. In particular, we apply it to four of the cell populations isolated in Guo et al. [15]: two cell types from a healthy mouse (GMP, Lin+) and two from a leukemic mouse (LGMP, LLin+), whose leukemic cells came from a MLL-AF9 fusion protein [25]. The most significant kinetic parameter changes are shown in Fig 7. Some of the genes profiled by Guo et al. [15] were found in fewer than 10 cells in one or more cell population, and these were excluded from kinetic parameter comparisons.

Some of the genes are enriched in a single cell population; for instance, TLR9, a factor whose expression influences the prognosis of leukemia [26], is found predominantly in the LLin+ cells, and appears to be regulated by *K*_*on*_. Most of the identified kinetic parameter changes were in *K*_*on*_ or a combination of *K*_*on*_ and *K*_*t*_. However, MUC13 and SIGLEC5 were predicted to have been regulated by only *K*_*t*_ and IL3RA was predicted to be regulated by *K*_*off*_. Interestingly, IL3RA is the only example where all methods predict changes in *K*_*off*_, which suggests that this is a promising gene to focus on in future research.

## Discussion

**Fig 8:**
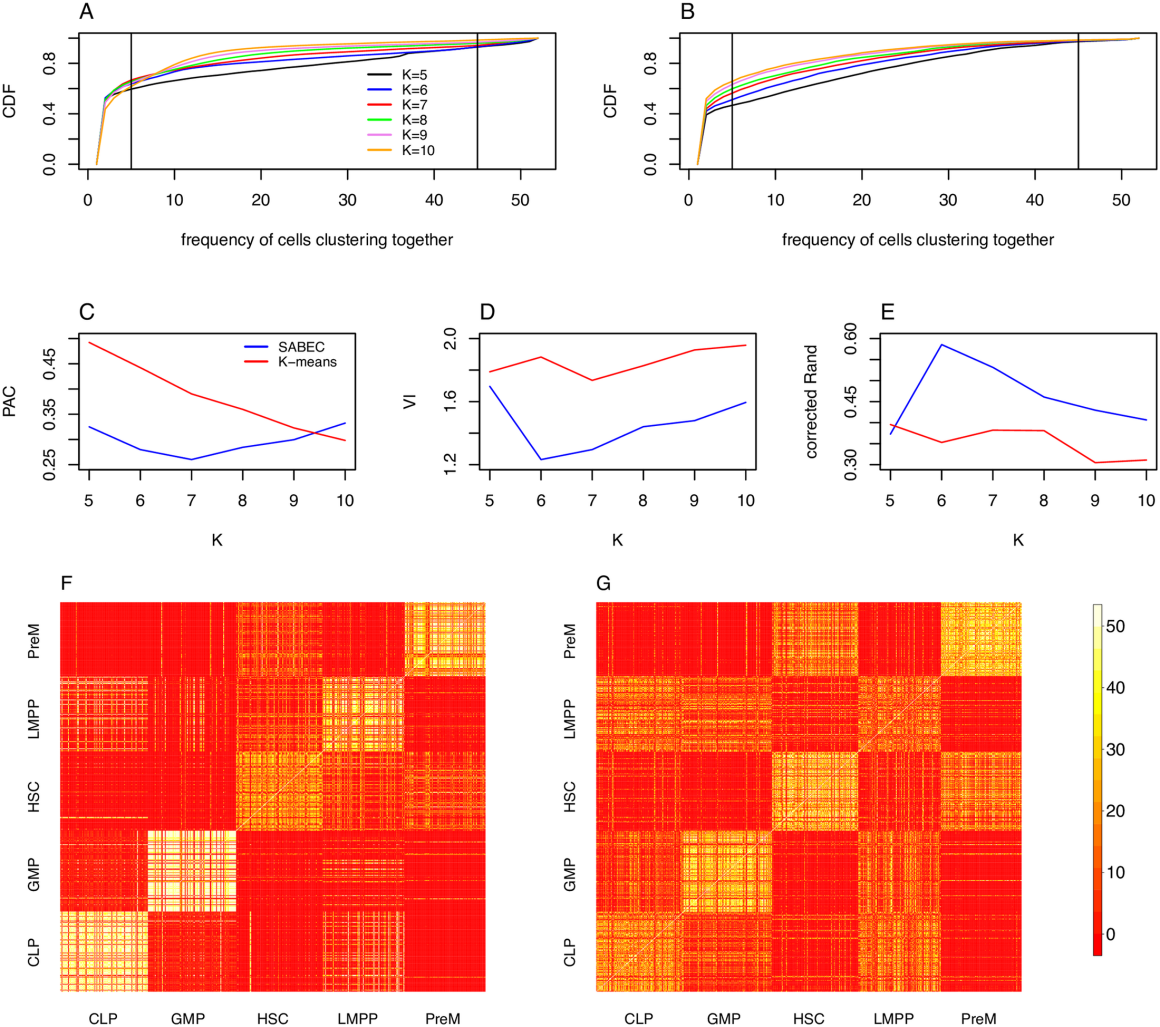
Consensus clustering comparison of SABEC and K-means. In the case of the Moignard dataset, SABEC is better than K-means at grouping cells by their FACS label. (A, B) For each pair of cells, we can count the number of times a pair of cells cluster together– a cumulative density function of these values, for K varying from 5 to 10, is shown in for SABEC (A) and K-means (B). Pairs of cells that are grouped together greater than 10% of the time and less than 90% of the time–represented by the vertical lines in (A) and (B)– are considered to be ambiguously clustered. (C) The proportion of cells that are ambiguously clustered (PAC) for SABEC and K-means is used to evaluate the robustness of the clustering approach. (D, E) Next we quantify the accuracy of the clusters, as compared to the labelled values (as per FACS). Specifically, this was done using the variable information (VI) (D) and the corrected Rand index (corrected Rand) (E). (F, G) The full consensus matrix, by cell type label according to FACS, for *K* = 6 are shown for SABEC (F) and K-means (G).

In this study, we exploit established mathematical models of bursting gene expression to develop a new pipeline for analyzing single cell qPCR data to more robustly cluster biological samples and provide insight into the mechanics of gene regulation. We apply these methods to study gene regulation in hematopoietic stem cell and progenitor populations. Even though single cell qPCR data can only provide snapshots of gene expression in a population of cells across different time points, we can infer the temporal dynamics of gene expression in these cells, and use this information to infer the population substructure (via SABEC) or regulatory mechanisms (via EPiK). This pipeline can be applied to study how genes are regulated during the natural process of differentiation or as cells progress into a diseased state. In addition, by manipulating the expression of a TF within its cell culture, we can infer its specific regulatory role. These algorithms perform well in simulated datasets (S1, S2, S6, S7, S8, S12, S13 and S14 Figs), performing better than similar computational tools (Figs 8, S3 and S5).

Instead of simply observing how much gene expression heterogeneity there is in a sample, it is now possible to predict the specific regulatory mechanisms that contributed to heterogeneity. Most crucially, this type of analysis can be done in a high-throughput manner.

In addition, commonplace clustering algorithms like K-means and hierarchical clustering are not meant to cluster data drawn from Poisson, negative binomal, and bimodal distributions, as is the case for single cell gene expression data. For instance, K-means performs best on data that comes from normal distributions with similar standard deviations. For this reason, it is critical to use a clustering algorithm that incorporates information about the shape of the gene expression distributions when analyzing single cell resolution datasets.

However, it is unclear how the cell cycle could influence our results, so future experiments ought to include cell cycle markers as controls [27]. In addition, our approach assumes that the gene expression distributions are close to equilibrium. Fortunately, Peccoud and Ycart [7] demonstrated that the two state model approaches the equilibrium distribution very rapidly (at an exponential rate), so this assumption likely holds. Other researchers have attempted to fit a multi-state model to data, instead of a two state model [28], but unless there is strong evidence for a more complex model, it is wise to use the simplest approach to avoid over-fitting the data. In the future, it may be possible to modify the model to detect cells that are in the process of transitioning between cell populations– for instance, these may be cells that have estimated kinetic parameters that are between two other populations. In addition, Teles et. al. [13] estimated the probability of transition between cell populations using machine learning methods, but this is beyond the scope of this paper.

One particularly useful application of this pipeline is the validation of assumptions used to model specific sub-networks of genes important in differentiation. Often, these models assume certain mechanisms by which the TFs influence one another. For instance, Narula et al. [29] constructed a mathematical model of a hematopoietic sub-network under the assumption that *K*_*off*_ was the parameter that is biologically regulated, in the absence of any experimental data. Instead of arbitrarily selecting a modeling strategy, we can now choose one that fits the data best. In addition, we have discovered that different TFs are regulated through different mechanisms in each stage of differentiation, implying that a single model of a gene network might not universally apply. Therefore, these strategies would allow us to make more biologically plausible models of gene subnetworks. Having a better understanding of gene regulation processes is important in order to learn how these are perturbed in disease, and also to develop protocols that produce desired cell types for cell therapy.

A modified version of this pipeline for RNA-seq data would be an important future development. The kinetic parameter estimation strategy and EPiK may be applied to single cell RNA-seq, as long as there is sufficient sequencing depth to capture the population-wide distribution of gene expression. Both these methods scale approximately linearly with the number of cells and genes under study, and could also be run in parallel for very large datasets. However, SABEC would not scale well with the large number of genes analysed in RNA-seq experiments, so alternative clustering approaches must be tested in an RNA-seq context.

The transcription process is a multi-step chemical reaction: chromatin must enter the correct state, TFs must bind in the right places, the general transcription machinery must be recruited and initiated, etc. It is currently impossible to distinguish the effects of all of these mechanisms in a high-throughput way. Our pipeline provides a first attempt to understand how the kinetic parameters underlying complex transcriptional processes influence heterogeneity within and across cell populations, through the analysis of single cell gene expression data.

## Methods

### Cell culture

HPC7 cells [21] were grown in suspension in Iscove’s Modified Eagle’s Medium (IMDM, Gibco) with 10% FCS, 10% stem cell factor-conditioned medium, 1% penicillin/streptomycin (Sigma) and 1.5 × 10^−4^ M monothioglycerol (MTG) at 37°C and 5% *CO*_2_. Cells were passaged every two days to maintain a concentration of 0.5 − 2 × 10^6^ cells/ml.

### Retroviral transduction

For knockdown experiments, shRNA fragments were cloned into pMSCV/LTRmiR30-PIG (pLMP, Open Biosystems): Luciferase (control, 5’ CACGTACGCGGAATACTTCGAA 3’, [30]), Gata2 (5’ CGCCGCCATTACTGTGAATATT 3’, [31]). For overexpression experiments, the mouse Gfi1 cDNA was inserted into pMSCV-ires-GFP (Addgene plasmid 20672), with the empty vector used as a control. Retrovirus was produced using the pCL-Eco Retrovirus Packaging Vector (Imgenex) in 293T cells.

HPC7 cells were infected with retrovirus by centrifugation at 800 xg at 32°C for 1.5 hours with 4 *μ*g/ml polybrene (Sigma), after which the retroviral supernatant was replaced with fresh media and cells were cultured as normal. Transduction efficiency was monitored by flow cytometry for GFP.

### Single cell gene expression analysis

Single GFP+ cells were sorted by FACS into individual wells of 96 well plates and single cell RT-qPCR was carried out as described previously [14]. Cells were captured 48 hours after retroviral transduction for Gfi1 overexpression and 72 hours after transduction for Gata2 knockdown (S1 Table).

### Estimation of kinetic parameters

Bursting gene expression can lead to a number of different distributions of gene expression in a population of cells (See Fig 1B), ranging from a bimodal distribution (when *K*_*on*_ and *K*_*off*_ are low) to a Poisson distribution (when *K*_*on*_ is much higher than *K*_*off*_) to an exponential decay-like distribution (when *K*_*off*_ is much higher than *K*_*on*_). The probability of having *x* mRNA molecules in a cell with kinetic parameters *K*_*on*_, *K*_*off*_ and *K*_*t*_ is given by the analytical solution developed by [11]:

$$P\left(x|K_{on},K_{off},K_{t}\right)=\frac{\Gamma \left(K_{on}+x\right)\Gamma \left(K_{on}+K_{off}\right)K_{t}^{x}}{\Gamma \left(x+1\right)\Gamma \left(K_{on}+K_{off}+x\right)\Gamma \left(K_{on}\right)}1F1\left(K_{on}+x,K_{on}+K_{off}+x,-K_{t}\right)$$ (1)

In this equation, 1*F*1 represents the confluent hypergeometric function of the first kind, a summation over an infinite series that is time intensive to compute. To improve our runtimes, we precomputed an extensive lookup table of values of *P*(*x*|*K*_*on*_, *K*_*off*_, *K*_*t*_). Let us say that we have *n* cells, each with an mRNA molecule count (for a particular gene) of *x*_*i*_, and let *X* = {*x*_0_, *x*_1_, …*x*_*n*_}. Given this list of mRNA counts from a semi-uniform population, we can assess the log likelihood of each possible set of parameters:

$$L\left(K_{on},K_{off},K_{t}|X\right)=\sum_{X}\left(\ln\left(P\left(x_{i}|K_{on},K_{off},K_{t}\right)\right)\right)$$ (2)

The set of kinetic parameters that has the maximum likelihood is chosen. However, it is important to note that some areas of the parameter space are more sensitive to parameter changes than others. For instance, [11] notes that at large values of *K*_*off*_ the equation of *P*(*x*|*K*_*on*_, *K*_*off*_, *K*_*t*_) approaches:

$$P^{\prime }\left(x|K_{on},K_{off},K_{t}\right)={\left(1+\frac{K_{t}}{K_{off}}\right)}^{-K_{on}}\frac{\Gamma (K_{on}+x)}{\Gamma \left(K_{on}\right)\Gamma \left(x+1\right)}{\left(\frac{\frac{K_{t}}{K_{off}}}{1+\frac{K_{t}}{K_{off}}}\right)}^{x}$$ (3)

**Fig 9:**
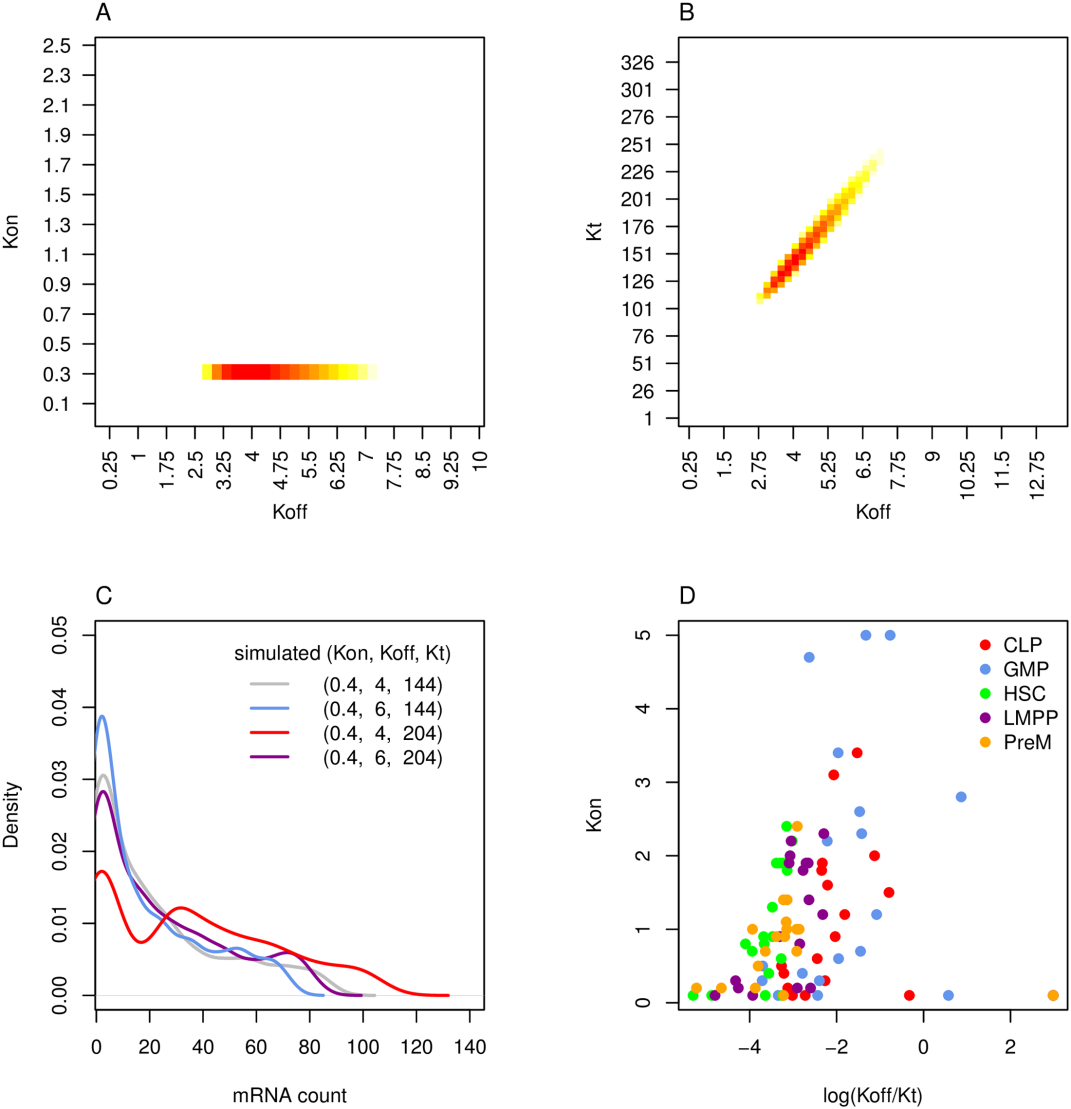
Properties of two-state model and kinetic parameter estimation strategy. Kinetic parameters control the shape of the gene expression distribution. Kinetic parameters are estimated by calculating the maximum likelihood from Eq 2. (A-B) In order to illustrate some properties of the two-state model, the likelihoods of various kinetic parameter sets were calculated for a toy example–specifically GFI1b gene expression data in hematopoietic stem cells (HSC). (A) For each possible *K*_*on*_ and *K*_*off*_ value, we calculated the maximum log likelihood across all possible *K*_*t*_ values. The overall log-maximum likelihood was 603.66 and only values that were within 0.5 of this value were coloured in, with the maximum value in dark red. (B) The maximum log likelihood of all possible *K*_*on*_ values were also calculated, as *K*_*off*_ and *K*_*t*_ were varied. There is a region in subfigure B where *K*_*off*_ and *K*_*t*_ can compensate for one another. (C) An example of parameter compensation was simulated: the original distribution (grey) has visible changes when *K*_*off*_ (blue) and *K*_*t*_ (red) are varied, but the distributions are barely distinguishable when both parameters are varied (purple). (D) Finally, we show our method’s estimates of *K*_*on*_ and *ln*(*K*_*off*_/*K*_*t*_) for each TF in each population of FACS cells.

This equation depends of the ratio of *K*_*t*_/*K*_*off*_ rather than *K*_*t*_ and *K*_*off*_ separately, which means that *K*_*off*_ and *K*_*t*_ are more difficult to distinguish as *K*_*off*_ increases. The practical implication of this observation is illustrated in Fig 9A and 9B, which depict the log likelihoods at different kinetic parameter values for GFI1b in HSC cells, with regions that have log likelihoods close to the peak value coloured in (specifically, within 0.5 of the maximum likelihood). Although there is only a narrow range of possible *K*_*on*_ values (A), there is a range of values where *K*_*off*_ and *K*_*t*_ can partially compensate for one another (B). A specific example is illustrated with simulated data in Fig 9C: while the original distribution of gene expression (grey) varies visibly when *K*_*off*_ (blue) or *K*_*t*_ (red) are varied, it is possible to change both variables (purple) and almost recover the original distribution. Furthermore, this analysis suggests that it would be difficult to incorporate any additional parameters into the system and find unique estimates for each of them.

In S1 Fig, we test the performance of this strategy of kinetic parameter estimation on simulated data, including artificial technical noise. Although *K*_*off*_ cannot be accurately identified when *K*_*off*_ > 5, the ratio of *K*_*off*_ to *K*_*t*_ is accurately predicted (S2 Fig). Our method also performs better than the Gibbs Sampling based approach developed by Kim and Marioni, 2012 (S3 Fig).

Next, we applied this maximum likelihood approach for kinetic parameter estimation on hematopoietic stem cell and progenitor populations (CLP, GMP, CLP, LMPP and PreM cells) from [14]. The values for *K*_*on*_ and *ln*(*K*_*off*_/*K*_*t*_) for the Moignard data are shown in Fig 9F. Note that many of the TFs were particularly chosen due to their importance in early differentiation of HSCs, so one would expect a lower level of gene expression (and therefore a higher *K*_*off*_/*K*_*t*_ ratio) in later stage progenitor populations such as CLP and GMP. There is a wide range of estimated kinetic parameter values across the TFs in each cell population; however, we need to ensure that these kinetic parameter estimates are not being skewed by mis-classified cells before we can evaluate whether these differences are statistically significant.

### Parameter range and look-up table

It is crucial to select a table of appropriate range and point density for applying to the experimental data. The criteria for selecting this table were: i) fewer than 5% of the experimental points were at the maximum parameter value for the *K*_*on*_ and *K*_*t*_ parameters. ii) *K*_*off*_ had to be high enough in order to include the parameter range in which only the ratio of *K*_*t*_ to *K*_*off*_ matter ii) the density of points was sufficient to minimise artifacts arising from the discretization of the parameter space.

Given these constraints, the range of parameters was chosen to be 0 < *K*_*on*_ < 5, 0 < *K*_*off*_ < 20 and 0 < *K*_*t*_ < 200 and possible mRNA counts as 0 < *x* < 200. The sampling density for *K*_*on*_ was every 0.1, for *K*_*off*_ every 0.4 and for *K*_*t*_ every 5 for a total of 395000 possible parameter sets.

We deposited the look-up table, the code used to generate the table in Mathematica and the code for calculating the maximum likelihood in R on Github: https://github.com/ezer/SingleCellPipelineOverview.

### Scaling qPCR data to mRNA counts

The main source of data analysed in this paper comes from Moignard et al. [14], and contains single cell qPCR data from five populations of hematopoietic stem cell and progenitor cells (CLP, GMP, HSC, LMPP, PreM), as determined by FACS, with approximately 124 cells in each population. The genes profiled by the qPCR include 18 TFs with crucial roles in cell fate, which were normalised to two “housekeeping” genes (PolII and Ubc), as described in Moignard et al. [14].

The outcome of a PCR experiment is a normalised *C*_*t*_ value, which relates to mRNA molecules (*x*) as follows:

$$x=int(b\cdot 2^{a-C_{t}})$$ (4)

where *a* and *b* are constants. For each TF, we chose *b*:

$$b_{TF}=\frac{x_{\max}}{2^{a-\min(X_{TF})}}$$ (5)

where *x*_*max*_ = 200 is the maximum number of mRNA pre-calculated in our lookup table and *X*_*TF*_ is the set of mRNA counts for the particular TF. This choice of *b*_*TF*_ stretches the values of *x* to have as wide a range as possible. It also removes the need to set an *a* parameter, since the equation for calculating *x* can be simplified to:

$$x=int(x_{max}\cdot 2^{\min\left(X_{TF}\right)-C_{t}})$$ (6)

It is important to note that while a different *b* parameter was chosen for each TF, this *b* factor is consistent across all five populations of cells, which is crucial for our later attempts to compare kinetic parameter values between cell populations. Even though the choice of *b* changes the absolute value of the kinetic parameters that are estimated, it has minimal effects on the strength of the linear correlation between the known and estimated values.

### Simulated Annealing for Bursty Expression Clustering (SABEC)

SABEC begins by assigning each cell to a random population 1 to K. Note that the total number of clusters K must be set at the start of the algorithm. Next, SABEC iteratively calculates the kinetic parameters of each of the K populations, and the cells are reassigned to new populations probabilistically.

In a standard Expectation Maximisation clustering algorithm, the probability of assigning a cell to a population is proportional to the ratio of the likelihoods of the cell coming from each population. Let the kinetic parameter set for a single population be *S*_*i*_(*g*) = (*K*_*on*_(*g*), *K*_*off*_(*g*), *K*_*t*_(*g*)), where *g* is the gene (between 1 and G) and *i* labels the population (between 1 and K), and let **x**(*g*) be the vector of mRNA counts for each gene, for a particular cell. The log likelihood for a certain population can be calculated as follows:

$$L\left(S_{i}|x\right)=\sum_{g=1}^{G}(\ln\left(P\left(x\left(g\right)|S_{i}\left(g\right)\right)\right)$$ (7)

If the sum of the likelihoods for each population is *L*_*tot*_, this value can be scaled as such:

$$L^{\prime }\left(S_{i}|x\right)=\frac{L(S_{i}|x)}{L_{tot}}$$ (8)

Since it would take a long time for this algorithm to converge if the clusters are close to one another, we add a temperature parameter, so that initially it is easy for cells to be assigned to different clusters, but it becomes harder and harder to swap clusters over time:

$$L^{\prime \prime }\left(S_{i}|x\right)=L^{\prime }{\left(S_{i}|x\right)}^{\tau t}$$ (9)

where *τ* is the temperature parameter and *t* is the iteration number of the algorithm (a counter that increments each time the cells are reassigned to new clusters). A cell is probabilistically assigned to a new cluster based on the relative values of *L*″(*S*_*i*_|**x**) for each population. The algorithm terminates when fewer than 5% of the cells swap clusters in an iteration, or after 100 iterations. S4 Fig shows how the accuracy of the algorithm depends of the temperature parameter, *τ*.

Since this algorithm is randomized and since it is possible for certain runs of the algorithm to fall into local optima, we run this algorithm 50 times and conduct a secondary consensus clustering step. In this step, a consensus matrix is produced, in which each cell of the matrix represents the number of times that two cells are found in the same cluster. A plot of the cumulative density function of the values of this matrix can help visualise the robustness of the clustering (See Fig 8A). For comparison and to show the necessity for SABEC, consensus clustering of K-means was also conducted (See Fig 8B).

Fig 8 shows the cumulative density functions for the number of times two cells cluster together for SABEC (*A*) and K-means (*B*). [32] determined that the most robust metric for comparing the consistency of consensus clustering methods is the Proportion Ambiguously Clustered (PAC) score, which is defined as the proportion of cell pairs that cluster together in 10% and 90% of the repeated runs of the algorithm. This corresponds to the proportion of cell pairs that lie between the vertical lines in the cumulative distribution functions in Fig 8(A) and 8(B). The PAC scores for SABEC and K-means are compared in C, illustrating that SABEC usually has more consistent outcomes than K-means, with the fewest ambiguously clustered cells at *K* = 7.

In addition, to estimate the accuracy of our method, we can assume that the expected cluster assignment (as determined by FACS) is our gold standard. By comparing the results of our clustering approach with the gold standard, we can estimate the accuracy of our method on the experimental data. To do this, we cluster each of our consensus matrices into five clusters using partitioning around medoids (PAM), a clustering approach similar to K-means (but more consistent since it uses data points as centres). We can then compare our results to the gold standard labels using metrics such as VI (See Fig 8D) and the corrected Rand index (See Fig 8E). SABEC performs better than K-means by these two metrics, with the estimated number of clusters equal to 6. The one exception is that the corrected Rand index suggests that K-means performs slightly better than SABEC when *K* = 5; however, SABEC provides substantially better outcomes at *K* = 6. Based on simulated datasets with values similar to the experimental data, we determined that these latter two methods provide more accurate estimates of the number of clusters than the PAC method, which can sometimes overestimate the appropriate number of clusters (S8 Fig).

Figs 8F and 2G compare the consensus matrices for *K* = 6 for SABEC (*F*) and K-means (*G*), with the cells sorted by their FACS-determined labels. SABEC results appear more consistent, with cells frequently clustering with other cells of the same type. In addition, any cells that are “misclassified” tend to cluster instead with cell populations of their parent or children populations. For instance, some HSC cells cluster with their child populations (PreM and LMPP), and some GMP and CLP cells cluster with their parent population (LMPP).

The R script for SABEC is available in Github: https://github.com/ezer/SingleCellPipelineOverview, including a sample input file to run in parallel on a Condor cluster, and appropriately merge the outputs.

### Parameters chosen for K-means and hierarchical clustering

The SABEC method was compared to hierarchical clustering and K-means approaches. The hierarchical clustering approach used was the default one associated with the heatmap function in R (Euclidean distance metric and complete clustering). The K-means approach used the default algorithm in R (Hartigan and Wong), but the maximum iterations was increased to 100 in order to be more comparable to the SABEC approach. K-means was repeated 50 times and an additional consensus clustering step was taken, in order to provide a fairer comparison to SABEC. Note that the input to both the hierarchical clustering and K-means algorithms were the normalised *C*_*t*_ values, while the input to SABEC is the scaled mRNA counts. These algorithm and distance metrics were chosen since they are the most commonly used. Other variations of hierarchical clustering and K-means were tested, but none of the results were significantly better or different than the ones shown.

### Estimation of Pairwise changes in Kinetics (EPiK)

The R script for determining which kinetic parameters vary across populations is available in Github: https://github.com/ezer/SingleCellPipelineOverview.

Three methods were tested on simulated datasets of genes with randomly selected kinetic parameters. We conducted these simulation tests for two different ranges of the kinetic parameters to illustrate that there is different sensitivity to parameter changes in different regions of the parameter space (S12, S13 and S14 Figs). These results suggest that changes in *K*_*off*_ cannot be accurately detected when *K*_*off*_ > 5. In addition, the methods often performs better when fewer kinetic parameters change at once (S12 and S17 Figs). The correctly identified simulated cases were those that have the largest magnitude of kinetic parameter change (S18 Fig) and had average kinetic parameter values closer to the origin, where the kinetic parameters are more accurately estimated (S19 Fig).

Two of the methods (MP and Subset methods), have continuous-valued outputs, and so a threshold must be set for determining whether or not a change in a kinetic parameter is likely significant. The thresholds were set to have approximately a 2% false positive rate, based on the *in silico* validation tests. The threshold values for the MP method when *K*_*off*_ < 5 are −6.3, −8.5 and −6.8 for *K*_*on*_, *K*_*off*_ and *K*_*t*_, and −4.9 and −6.0 for *K*_*on*_ and *K*_*t*_ when *K*_*off*_ > 5. For the subset method, the thresholds are 0.77, 0.91 and 0.86 (*K*_*on*_, *K*_*off*_ and *K*_*t*_, respectively) when *K*_*off*_ < 5, and 0.681 and 0.870 (*K*_*on*_ and *K*_*t*_) when *K*_*off*_ > 5.

#### BICs-based method

There are eight possible parameter sets that could have varied across the two populations of cells for each TF (no parameters adjusted, all parameters adjusted, three cases with one parameter adjusted and three cases with two parameters adjusted). It is possible to calculate the maximum likelihood of each scenario occurring. Let us assume we have a matrix *L*_*p*,*m*_ that lists the likelihoods for each population of cells *p* = 1, 2 and parameter set in the lookup table *m* = 1 to *356,000*. The maximum likelihood score when no parameter is being adjusted is:

$$L_{none}=\max_{m}\left(L_{1,m}+L_{2,m}\right)$$ (10)

Meanwhile, the maximum likelihood score when all the parameters are being adjusted is:

$$L_{all}=\max_{m}\left(L_{1,m}\right)+\max_{m}\left(L_{2,m}\right)$$ (11)

Clearly, *L*_*all*_ will always be greater than or equal to *L*_*none*_. Therefore, we must adjust these likelihoods to take into account that fact that they involve different numbers of parameters being adjusted. This is done as follows:

$${BIC}_{i}=N_{i}\left(\log\left(2n\right)-\log\left(2\pi \right)\right)-2L_{i}$$ (12)

Where *i* indices the set of parameters being adjusted, *N*_*i*_ is the number of parameters presumed adjusted, *n* is the number of cells and *L*_*i*_ is the likelihoods, for example, *L*_*none*_ or *L*_*all*_ calculated above.

#### Marginal probability-based method

To calculate an MP score for *K*_*on*_, let us make two matrices *A*(*i*, *j*) and *B*(*i*, *j*), one for each of the two populations of cells we are trying to compare. Each contains *L*(*K*_*on*_, *K*_*off*_, *K*_*t*_|*X*), with *i* indexing the unique set of *K*_*on*_ values (with *i* = 1 to *I*) and *j* indexing a unique set of (*K*_*off*_, *K*_*t*_) pairs (with *j* = 1 to *J*).

$$A_{tot}=\log\left(\sum_{i=1}^{I}\sum_{j=1}^{J}e^{A(i,j)}\right)$$ (13)

$$A^{\prime }\left(i\right)=\log\left(\sum_{j=1}^{J}e^{A\left(i,j\right)-A_{tot}}\right)$$ (14)

*B*′(*i*) can be calculated in the same way as *A*′(*i*).

$$MP_{K_{on}}=\log\left(\sum_{i=1}^{I}e^{\left(A^{\prime }\left(i\right)+B^{\prime }\left(i\right)\right)}\right)$$ (15)

However, it is important to implement this strategy in such a way as to minimize the rounding errors caused by taking the exponent of negative number with large magnitude. This can be accomplished thanks to the following statement:

$$\log\left(e^{a}+e^{b}\right)=\log\left(e^{a-x}+e^{b-x}\right)+x$$ (16)

By choosing *x* to be close to *a* or *b*, it is possible to avoid such errors. This strategy is employed in all the calculations above involving summing exponents.

#### Subsetting method

In the third method, we repeatedly subsample 25% of the cells (100 times) and estimate the kinetic parameters for each subset. For each of the three options for the kinetic parameter, we calculate the absolute distance between the cumulative density functions of the two distributions, aka the Kolmogorov-Smirnov Statistic (S14 Fig). Note that we cannot use the p-values from the Kolmogorov-Smirnov test, because we re-sample points many times, so this statistic would be biased towards lower p-values. The threshold for the K-S statistic that was deemed significant was the one with a 2% false positive rate. Changing the size of the subsamples only had a small effect on the accuracy of this strategy (S16 Fig).

## Supporting Information

S1 Fig Parameter estimates for simulated data. Subfigures A-C show parameter estimates for simulated parameter sets randomly drawn from 0 < *K*_*on*_ < 5, 0 < *K*_*off*_ < 10 and 0 < *K*_*t*_ < 600, for *K*_*on*_ (A), *K*_*off*_ (B) and *K*_*t*_ (C). Subfigures D-F show the same, but with 10 < *K*_*off*_ < 20, also for *K*_*on*_ (D), *K*_*off*_ (E) and *K*_*t*_ (F). Note that the known and estimated kinetic parameter values are all linearly correlated, except for *K*_*off*_ when it is greater than approximately 5.
(TIF)

S2 Fig Estimates of *K*_*t*_/*K*_*off*_ ratios in simulated datasets. These figures compare known and estimates *K*_*t*_/*K*_*off*_ ratios for the same datasets depicted in S1 Fig with (A) depicting simulations where 0 < *K*_*off*_ < 10 and (B) depicting simulations where 10 < *K*_*off*_ < 20. In both cases, the known and estimated ratio of *K*_*t*_/*K*_*off*_ is linearly correlated.
(TIF)

S3 Fig Parameter estimation with Gibb’s Sampling. The kinetic parameters of the simulated datasets were estimated using the Gibb’s Sampling approach introduced by [12]. This method was designed for RNA-seq, so the gene *length* is a required input, but 10,000bp was included for all genes and all the mRNA counts were multiplied by this value. *K*_*on*_ is always between 0 and 5, and *K*_*t*_ is always between 0 and 600, and 90% of the mRNA molecules are randomly removed. Subfigures *A-C* are for simulations with 0 < *K*_*off*_ < 2, *D-F* are for 0 < *K*_*off*_ < 10 and *G-I* are for 10 < *K*_*off*_ < 20. *K*_*on*_ is estimated in subfigures *A, D, G*, *K*_*off*_ is estimated in subfigures *B, E, H* and *K*_*t*_ is estimated in subfigures *C, F, I*.
(TIF)

S4 Fig Choosing the temperature parameter. SABEC requires a choice of a temperature parameter (we chose 10 for most of this paper), which speeds convergence of the algorithm. 100 simulation datasets were clustered with SABEC (each repeated 50 times). The y-axes of *A* shows the average number of iterations before convergence across these 50 repeats. Convergence is defined as the number of iterations of the algorithm until fewer than 5% of the cells swapping clusters, but it is capped at a maximum of 100 iterations. The grey shaded area represents the full range of average values across all 100 simulated datasets and the black line represents the overall average number of iterations. This subfigure illustrates that the larger the temperature, the quicker the algorithm converges. Subfigures *B,C* illustrate how temperature influences the accuracy of the algorithm, as per the average variable information (VI) and corrected Rand index across all 100 simulated datasets. Both these metrics illustrate that the higher the temperature, the less accurate the algorithm. The aberration when the temperature parameter has a value of 6 comes from the fact that the number of iterations is capped at 100, so in some cases the algorithm did not fully converge. Note that we choose a temperature of 10 elsewhere in this paper.
(TIF)

S5 Fig Hierarchical clustering of hematopoietic stem cell and progenitor populations. Here are the results of the hierarchical clustering of the normalised qPCR data, color coded the same way as Fig 4.
(TIF)

S6 Fig Example results of SABEC for simulated dataset. Out of the 100 simulated datasets that were generated, three examples are illustrated here, with their consensus matrices shown in subfigures *A-C* and the clustered heatmaps of these in *D-F*. These include the dataset that had the worst robustness by PAC score (*A,D*), the best robustness (*C, F*) and a randomly selected third example (*B,E*). The colours in *D-F* are blue for CLP, red for GMP, green for HSC, purple for LMPP and orange for PreM. Note that the clustering is more robust than the experimental dataset. In fact, manual inspection of 100 clusterings found no example of HSC being split into two clusters, while GMP/CLP being clustered together (the scenario observed in the experimental dataset), suggesting that the subdivision of HSC into two clusters is probably not an artifact of the SABEC method.
(TIF)

S7 Fig SABEC applied to simulated datasets with different numbers of genes and cells. First, we generated a list of 100 parameter sets that were randomly selected from a normal distribution around the experimentally determined kinetic parameter values, with a standard deviation equal to 5% of the parameter range in our look-up table (specifically, 0.25, 1 and 10, for *K*_*on*_, *K*_*off*_ and *K*_*t*_ respectively). This created a kinetic parameter distribution that was similar to the distribution estimated for the experimental data by [14]. For each simulated dataset, we randomly selected kinetic parameter sets from this list, varying the number of genes and the number of cells, but keeping the number of populations at 5. For each choice of number of genes and number of cells, we repeated this procedure with 5 different simulated datasets. In each case, the SABEC method was used to cluster the dataset (including the consensus clustering step). Subfigure *A* illustrates the variable information (VI) and *B* shows the corrected Rand index. Subfigure *C* shows the average number of iterations of SABEC until convergence.
(TIF)

S8 Fig Estimating K for simulated datasets. We wished to determine how reliable PAC, VI and the corrected Rand index were at predicting the correct number of clusters when interpreting SABEC data. For instance, it could be that the SABEC method consistently causes there to appear to be more clusters than there actually are, which could mean that the division of HSC into two clusters is spurious. After predicting the number of clusters in the simulated datasets, with each of the 100 resulting curves drawn for PAC (*A*), VI (*B*) and the corrected Rand index (*C*), we see that PAC often predicts a spurious cluster, but VI and the correct Rand index do not. This is one of the reasons we selected *K* = 6, trusting VI and the corrected Rand index more than the PAC score.
(TIF)

S9 Fig HSC subpopulations are separated before CLP/GMP populations separate. Here we show clustered heatmaps of HSC (green), LMPP (purple) and PreM (orange) cells (*A-C*) and LMPP (purple), CLP (blue) and GMP (red) (*D-F*) for the number of clusters *K* = 5 (*A,D*), *K* = 6 (*B, E*) and *K* = 7 (*C,F*). Specifically note how the distinction between the two subpopulations of HSC is defined even in *K* = 5, but CLP and GMP are not distinguished until *K* = 6.
(TIF)

S10 Fig Comparison of parameter predictions before and after pruning. Cells that did not cluster well with other cells of their labelled population were removed in a pruning step, because these cells may not have uniform gene expression bursting kinetics compared to the other cells in their population. However, we were worried whether this would create a consistent bias in the parameter estimates. The line of best fit is designated as the red dashed line. Subfigures *A-D* compare the original estimates of the kinetic parameters on the complete dataset with the parameter estimates for the pruned datasets in the [14] data, for *K*_*on*_ (*A*), *K*_*off*_(*B*), *K*_*t*_(*C*) and *K*_*t*_/*K*_*off*_(*D*). Subfigures *E-H* also compare the parameter estimate changes in the complete and pruned datasets, but for the 100 simulated datasets. Note that removing outliers does bias the kinetic parameter estimates, in the same direction as observed in the experimental data, but to a much smaller extent. In particular, estimated *K*_*on*_ parameters increase and *K*_*off*_ and *K*_*t*_ values decrease after the pruning procedure, in both cases.
(TIF)

S11 Fig Bias in kinetic parameter predictions can be caused my mixed cell types. Another potential cause of the change in kinetic parameter estimates after pruning observed in Fig 4 is that the pruning procedure correctly removed cells that did not have homogenous bursting kinetics. In this figure, we compare the kinetic parameter estimates when cell populations are mixed with cells of a different type to the kinetic parameter estimates when the cell populations are pure. For each of the 100 simulated datasets, 10 cells of each population were re-labelled as coming from an incorrect cell type. A comparison of the estimated kinetic parameters in the mixed and pure cell populations are shown for CLP (*A*–*C*), GMP (*D*–*F*), HSC (*G*–*I*), LMPP (*J*–*L*) and PreM (*M*–*O*). The red line designates the line-of-best fit. Note that the bias observed in S10 Fig is seen prominently here: *K*_*on*_ estimates are higher in mixed cell populations, while *K*_*off*_ and *K*_*t*_ estimates are lower, and the magnitude of the change is much more consistent with that seen in the experimental data. Nevertheless, we cannot neglect the fact that removing false negative cells can also bias the kinetic parameter estimates.
(TIF)

S12 Fig BIC results for simulated datasets. 800 simulated datasets with *K*_*off*_ < 5 (*A*) and 800 simulated datasets with 5 < *K*_*off*_ < 10 (*B*) were generated, with 100 examples of each possible type of kinetic parameter set change (x-axis). For each of these, BIC was used to predict the kinetic parameter change, as shown by the colors in the stacked bar plot. Grey represents the case that none of the parameters change and black represents the case that all the kinetic parameters change. The primary colors (red, blue, yellow) correspond with the case of one kinetic parameter changes (*K*_*on*_, *K*_*off*_ and *K*_*t*_, respectively). The secondary colors correspond with the cases where two kinetic parameters change, with purple as *K*_*on*_ and *K*_*off*_, orange as *K*_*on*_ and *K*_*t*_ and green as *K*_*off*_ and *K*_*t*_. Clearly, BIC enriches for the correct set of parameters, although it is a bit conservative when more than one kinetic parameter is varied. Note that when *K*_*off*_ > 5, *K*_*off*_ is often mislabeled as *none* and sometimes as *K*_*t*_.
(TIF)

S13 Fig MP results for simulated datasets. These are the ROC curves for *K*_*off*_ < 5 (*A*–*C*) and *K*_*off*_ > 5 (*D*–*F*), with the area under the curve (AUC) listed for each figure. Based on the high values of AUC, this method is predictive for *K*_*on*_ and *K*_*t*_, but less so for *K*_*off*_. Note that predictive power for *K*_*off*_ completely disappears at higher values, although it does not seem to substantially increase *K*_*t*_ false positive rates.
(TIF)

S14 Fig Subset results for simulated datasets. In black are the ROC curves for *K*_*off*_ < 5 (*A*–*C*) and *K*_*off*_ > 5 (*D*–*F*), with the area under the curve (AUC) listed for each figure. The corresponding MP ROC curves are drawn in grey for comparison. The results are fairly similar, although notably the results are worse than MP for *K*_*t*_ when *K*_*off*_ > 5.
(TIF)

S15 Fig Distribution of kinetic parameter estimates under different methods. These pie charts display the proportion of different kinetic parameter combinations deemed to have varied for each TF between pairs of populations of cells connected by lines in Fig 4(Bi). The colors designate which kinetic parameter was adjusted, yellow for *K*_*t*_, red for *K*_*on*_, blue for *K*_*off*_, orange for *K*_*t*_ and *K*_*on*_, green for *K*_*t*_ and *K*_*off*_, purple for *K*_*on*_ and *K*_*off*_, grey for no changes and black for all parameters. Subfigures *A*–*B* are for BIC, *C*–*D* for marginal probability (MP) and *E*–*F* for the subset method (Sub). Each of the methods was applied to the dataset where potential outliers were pruned (*A*, *C* and *E*) and the complete datasets before the pruning step (*B*, *D* and *F*).
(TIF)

S16 Fig Varying subsample size. In order to determine whether the specificity and sensitivity of the method depends significantly on the size of the subsamples, we re-ran the subsample method, but sample half the cells (62) instead of a quarter of the cells, as shown in S14 Fig. The grey line still represents the ROC curve for the MP method. The AUC scores are similar across both subset sizes.
(TIF)

S17 Fig How assumptions about frequency of parameter changes influence accuracy of MP and subset method. The simulated datasets which were used to test the accuracy of these three methods have an equal number of examples from each of the eight possible sets of parameters that can change. However, we saw in S12 Fig that BIC misses many true positives when more than one kinetic parameter varies. We wondered whether the accuracy of the MP and subset methods are influenced by the assumptions of the number of kinetic parameters that were varied. The black lines and corresponding black AUC values consider the 400 simulated datasets where only 0 or 1 kinetic parameters are varied, while the blue lines and blue AUC values consider the other 400 simulated datasets, where 2 or 3 kinetic parameters are varied. Subfigures *A*–*C* show the results for the MP method and *D*–*E* show the results for the subset method. In both cases, only the examples when with *K*_*off*_ < 5 are shown. Estimates for *K*_*on*_ appear to be similar under both assumptions, but the estimates for *K*_*off*_ and *K*_*t*_ drop slightly when there are more kinetic parameters that vary (the blue line).
(TIF)

S18 Fig Influence of magnitude of kinetic parameter changes on sensitivity of the intersection method. This histogram shows the full distribution of the magnitude of kinetic parameter variation among simulated datasets in which that kinetic parameter varied (positives) for *K*_*on*_ (*A*), *K*_*off*_ (B) and *K*_*t*_ (*C*). The samples that were correctly identified by the intersection of the three methods (the true positives) are shaded dark grey, while the false negatives are light grey. Note that the proportion of true positive values increases as the magnitude of the kinetic parameter change increases.
(TIF)

S19 Fig Influence of average kinetic parameter values on sensitivity of the intersection method. This figure is parallel to S18 Fig, except instead of subtracting the two kinetic parameters assigned to the pair of populations, these two values are *averaged*. For *K*_*on*_ (A), *K*_*off*_ (B) and to a lesser extent *K*_*t*_ (C), there is a higher rate of detection when the values are closest to the origin, the region where the kinetic parameters are more easily discernible.
(TIF)

S20 Fig Effectiveness of experiments to up regulate GFI1 and down regulate GATA2. These histograms depict the normalised Ct values for the GATA2 down regulation experiment (*A*), the GATA2 control HPC7 cells (*B*), the GFI1 up regulation experiment (*C*), and the GFI1 control HPC7 cells (*D*). The values where Ct is 15 are cells where there was no visible expression of the gene. GFI1 up regulation was successful for all of the cell, but GATA2 down regulation only slightly decreased its overall expression compared to the controls.
(TIF)

S1 Table Single cell gene expression dataset. This table includes the raw single cell gene cell qPCR data from HPC7 cells for the Gfi1 overexpression and Gata2 knockdown experiments.
(XLSX)

## References

[1] Ko M, Nakauchi H, Takahashi N. The dose dependence of glucocorticoid-inducible gene expression results from changes in the number of transcriptionally active templates. The EMBO journal. 1990;9(9):2835 2167833 10.1002/j.1460-2075.1990.tb07472.x PMC551995

[2] Yu J, Xiao J, Ren X, Lao K, Xie XS. Probing gene expression in live cells, one protein molecule at a time. Science. 2006;311(5767):1600–1603. 10.1126/science.1119623 16543458

[3] Muramoto T, Cannon D, Gierlinski M, Corrigan A, Barton GJ, Chubb JR. Live imaging of nascent RNA dynamics reveals distinct types of transcriptional pulse regulation. Proc Natl Acad Sci U S A. 2012 5;109(19):7350–7355. 10.1073/pnas.1117603109 22529358 PMC3358836

[4] Suter DM, Molina N, Gatfield D, Schneider K, Schibler U, Naef F. Mammalian genes are transcribed with widely different bursting kinetics. Science. 2011 4;332(6028):472–474. 10.1126/science.1198817 21415320

[5] Dar RD, Razooky BS, Singh A, Trimeloni TV, McCollum JM, Cox CD, et al Transcriptional burst frequency and burst size are equally modulated across the human genome. Proceedings of the National Academy of Sciences. 2012;109(43):17454–17459. 10.1073/pnas.1213530109 PMC3491463 23064634

[6] Ko MS. A stochastic model for gene induction. Journal of Theoretical Biology. 1991;153(2):181–194. 10.1016/S0022-5193(05)80421-7 1787735

[7] Peccoud J, Ycart B. Markovian modeling of gene-product synthesis. Theoretical population biology. 1995;48(2):222–234. 10.1006/tpbi.1995.1027

[8] Brown CR, Mao C, Falkovskaia E, Jurica MS, Boeger H. Linking stochastic fluctuations in chromatin structure and gene expression. PLoS Biol. 2013;11(8):e1001621 10.1371/journal.pbio.1001621 23940458 PMC3735467

[9] Dadiani M, van Dijk D, Segal B, Field Y, Ben-Artzi G, Raveh-Sadka T, et al Two DNA-encoded strategies for increasing expression with opposing effects on promoter dynamics and transcriptional noise. Genome research. 2013;23(6):966–976. 10.1101/gr.149096.112 23403035 PMC3668364

[10] Pennington KL, Marr SK, Chirn GW, Marr MT. Holo-TFIID controls the magnitude of a transcription burst and fine-tuning of transcription. Proceedings of the National Academy of Sciences. 2013;110(19):7678–7683. 10.1073/pnas.1221712110 PMC3651496 23610421

[11] Raj A, Peskin CS, Tranchina D, Vargas DY, Tyagi S. Stochastic mRNA synthesis in mammalian cells. PLoS Biol. 2006 10;4(10):e309 10.1371/journal.pbio.0040309 17048983 PMC1563489

[12] Kim JK, Marioni JC. Inferring the kinetics of stochastic gene expression from single-cell RNA-sequencing data. Genome Biol. 2013 1;14(1):R7 10.1186/gb-2013-14-1-r7 23360624 PMC3663116

[13] Teles J, Pina C, Ohlsson M, Enver T, Peterson C. Transcriptional regulation of lineage commitment–a stochastic model of cell fate decisions. PLoS Comput Biol. 2013 8;9(8):e1003197 10.1371/journal.pcbi.1003197 23990771 PMC3749951

[14] Moignard V, Macaulay IC, Swiers G, Buettner F, Schütte J, Calero-Nieto FJ, et al Characterization of transcriptional networks in blood stem and progenitor cells using high-throughput single-cell gene expression analysis. Nat Cell Biol. 2013 4;15(4):363–372. 10.1038/ncb2709 23524953 PMC3796878

[15] Guo G, Luc S, Marco E, Lin TW, Peng C, Kerenyi MA, et al Mapping cellular hierarchy by single-cell analysis of the cell surface repertoire. Cell Stem Cell. 2013 10;13(4):492–505. 10.1016/j.stem.2013.07.017 24035353 PMC3845089

[16] Wilson NK, Foster SD, Wang X, Knezevic K, Schütte J, Kaimakis P, et al Combinatorial transcriptional control in blood stem/progenitor cells: genome-wide analysis of ten major transcriptional regulators. Cell stem cell. 2010;7(4):532–544. 10.1016/j.stem.2010.07.016 20887958

[17] Arinobu Y, Mizuno Si, Chong Y, Shigematsu H, Iino T, Iwasaki H, et al Reciprocal activation of GATA-1 and PU. 1 marks initial specification of hematopoietic stem cells into myeloerythroid and myelolymphoid lineages. Cell stem cell. 2007;1(4):416–427. 10.1016/j.stem.2007.07.004 18371378

[18] Shin JY, Hu W, Naramura M, Park CY. High c-Kit expression identifies hematopoietic stem cells with impaired self-renewal and megakaryocytic bias. The Journal of experimental medicine. 2014;211(2):217–231. 10.1084/jem.20131128 24446491 PMC3920569

[19] Chyla BJ, Moreno-Miralles I, Steapleton MA, Thompson MA, Bhaskara S, Engel M, et al Deletion of Mtg16, a target of t (16; 21), alters hematopoietic progenitor cell proliferation and lineage allocation. Molecular and cellular biology. 2008;28(20):6234–6247. 10.1128/MCB.00404-08 18710942 PMC2577421

[20] Li Y, Okuno Y, Zhang P, Radomska HS, Chen Hm, Iwasaki H, et al Regulation of the PU. 1 gene by distal elements. Blood. 2001;98(10):2958–2965. 10.1182/blood.V98.10.2958 11698277

[21] Pinto do O P, Kolterud A, Carlsson L. Expression of the LIM-homeobox gene LH2 generates immortalized steel factor-dependent multipotent hematopoietic precursors. EMBO J. 1998 10;17(19):5744–5756. 10.1093/emboj/17.19.5744 9755174 PMC1170902

[22] van der Meer LT, Jansen JH, van der Reijden BA. Gfi1 and Gfi1b: key regulators of hematopoiesis. Leukemia. 2010 11;24(11):1834–1843. 10.1038/leu.2010.195 20861919

[23] Sánchez-Castillo M, Ruau D, Wilkinson AC, Ng FSL, Hannah R, Diamanti E, et al CODEX: a next-generation sequencing experiment database for the haematopoietic and embryonic stem cell communities. Nucleic Acids Res. 2015 1;43(Database issue):D1117–D1123. 10.1093/nar/gku895 25270877 PMC4384009

[24] Mollica LR, Crawley JTB, Liu K, Rance JB, Cockerill PN, Follows GA, et al Role of a 5’-enhancer in the transcriptional regulation of the human endothelial cell protein C receptor gene. Blood. 2006 8;108(4):1251–1259. 10.1182/blood-2006-02-001461 16627757

[25] Krivtsov AV, Twomey D, Feng Z, Stubbs MC, Wang Y, Faber J, et al Transformation from committed progenitor to leukaemia stem cell initiated by MLL–AF9. Nature. 2006;442(7104):818–822. 10.1038/nature04980 16862118

[26] Efremov DG, Bomben R, Gobessi S, Gattei V. TLR9 signaling defines distinct prognostic subsets in CLL. Front Biosci (Landmark Ed). 2013;18:371–386. 10.2741/4108 23276930

[27] Buettner F, Natarajan KN, Casale FP, Proserpio V, Scialdone A, Theis FJ, et al Computational analysis of cell-to-cell heterogeneity in single-cell RNA-sequencing data reveals hidden subpopulations of cells. Nat Biotechnol. 2015 2;33(2):155–160. 10.1038/nbt.3102 25599176

[28] Zhou T, Zhang J. Analytical results for a multistate gene model. SIAM Journal on Applied Mathematics. 2012;72(3):789–818. 10.1137/110852887

[29] Narula J, Smith AM, Gottgens B, Igoshin OA. Modeling reveals bistability and low-pass filtering in the network module determining blood stem cell fate. PLoS Comput Biol. 2010 5;6(5):e1000771 10.1371/journal.pcbi.1000771 20463872 PMC2865510

[30] Bot I, Guo J, Van Eck M, Van Santbrink PJ, Groot PH, Hildebrand RB, et al Lentiviral shRNA silencing of murine bone marrow cell CCR2 leads to persistent knockdown of CCR2 function in vivo. Blood. 2005;106(4):1147–1153. 10.1182/blood-2004-12-4839 15886324

[31] Huang Z, Dore LC, Li Z, Orkin SH, Feng G, Lin S, et al GATA-2 reinforces megakaryocyte development in the absence of GATA-1. Mol Cell Biol. 2009 9;29(18):5168–5180. 10.1128/MCB.00482-09 19620289 PMC2738300

[32] Senbabaoglu Y, Michailidis G, Li JZ. Critical limitations of consensus clustering in class discovery. Sci Rep. 2014;4:6207 10.1038/srep06207 25158761 PMC4145288

[33] Islam S, Zeisel A, Joost S, La Manno G, Zajac P, Kasper M, et al Quantitative single-cell RNA-seq with unique molecular identifiers. Nature methods. 2014;11(2):163–166. 10.1038/nmeth.2772 24363023

[34] Wu AR, Neff NF, Kalisky T, Dalerba P, Treutlein B, Rothenberg ME, et al Quantitative assessment of single-cell RNA-sequencing methods. Nature methods. 2014;11(1):41–46. 10.1038/nmeth.2694 24141493 PMC4022966

