# Supplementary figures and images for: Determining Physical Mechanisms of Gene Expression Regulation from Single Cell Gene Expression Data
Source: PLoS Comput Biol. 2016 Aug 23;12(8):e1005072. doi: 10.1371/journal.pcbi.1005072 (PMC4995004; doi:10.1371/journal.pcbi.1005072)

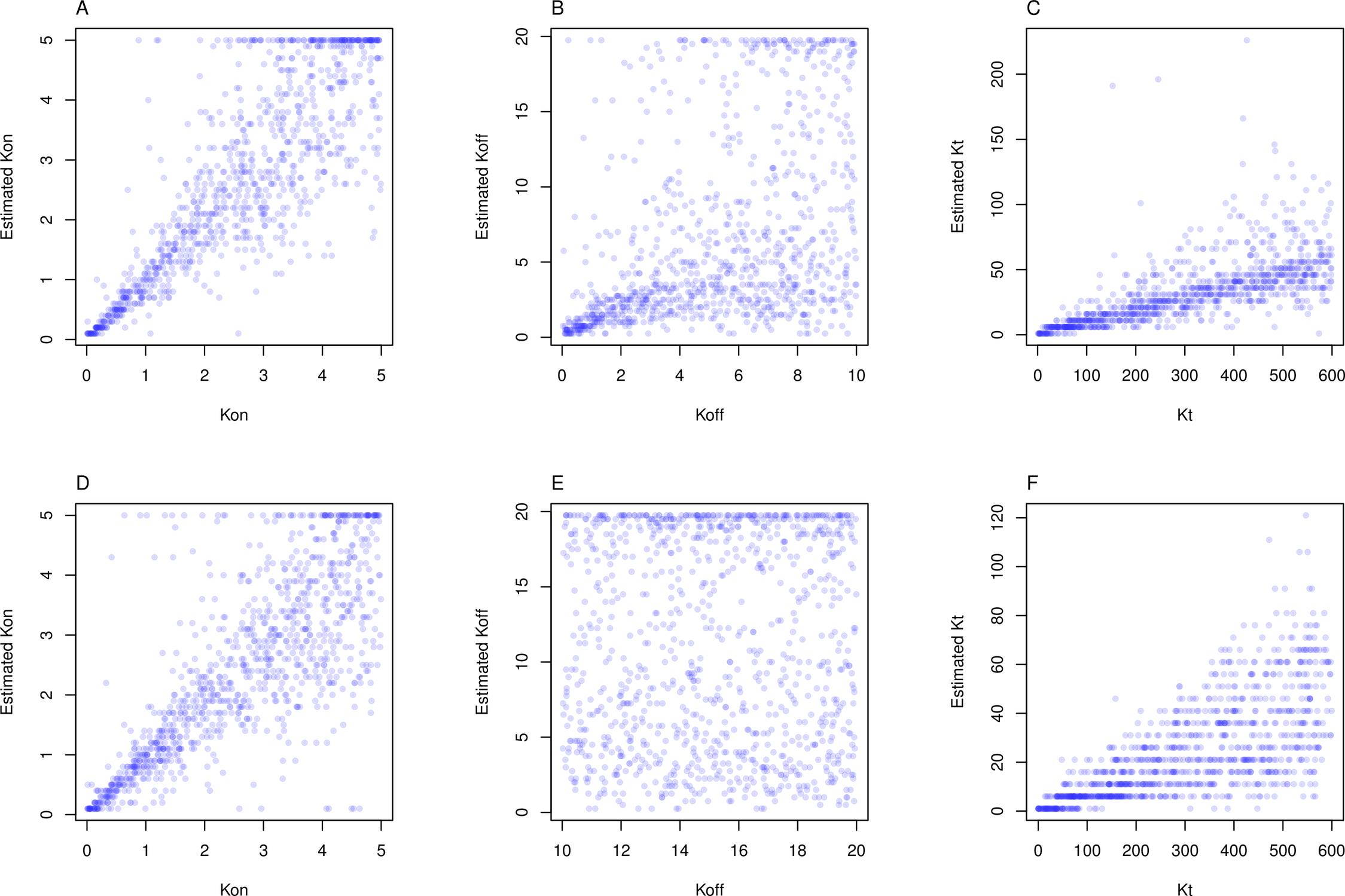

Supplement: S1 Fig — Subfigures A-C show parameter estimates for simulated parameter sets randomly drawn from 0 < Kon < 5, 0 < Koff < 10 and 0 < Kt < 600, for Kon (A), Koff (B) and Kt (C). Subfigures D-F show the same, but with 10 < Koff < 20, also for Kon (D), Koff (E) and Kt (F). Note that the known and estimated kinetic parameter values are all linearly correlated, except for Koff when it is greater than approximately 5. (TIF) [file pcbi.1005072.s001.tif]

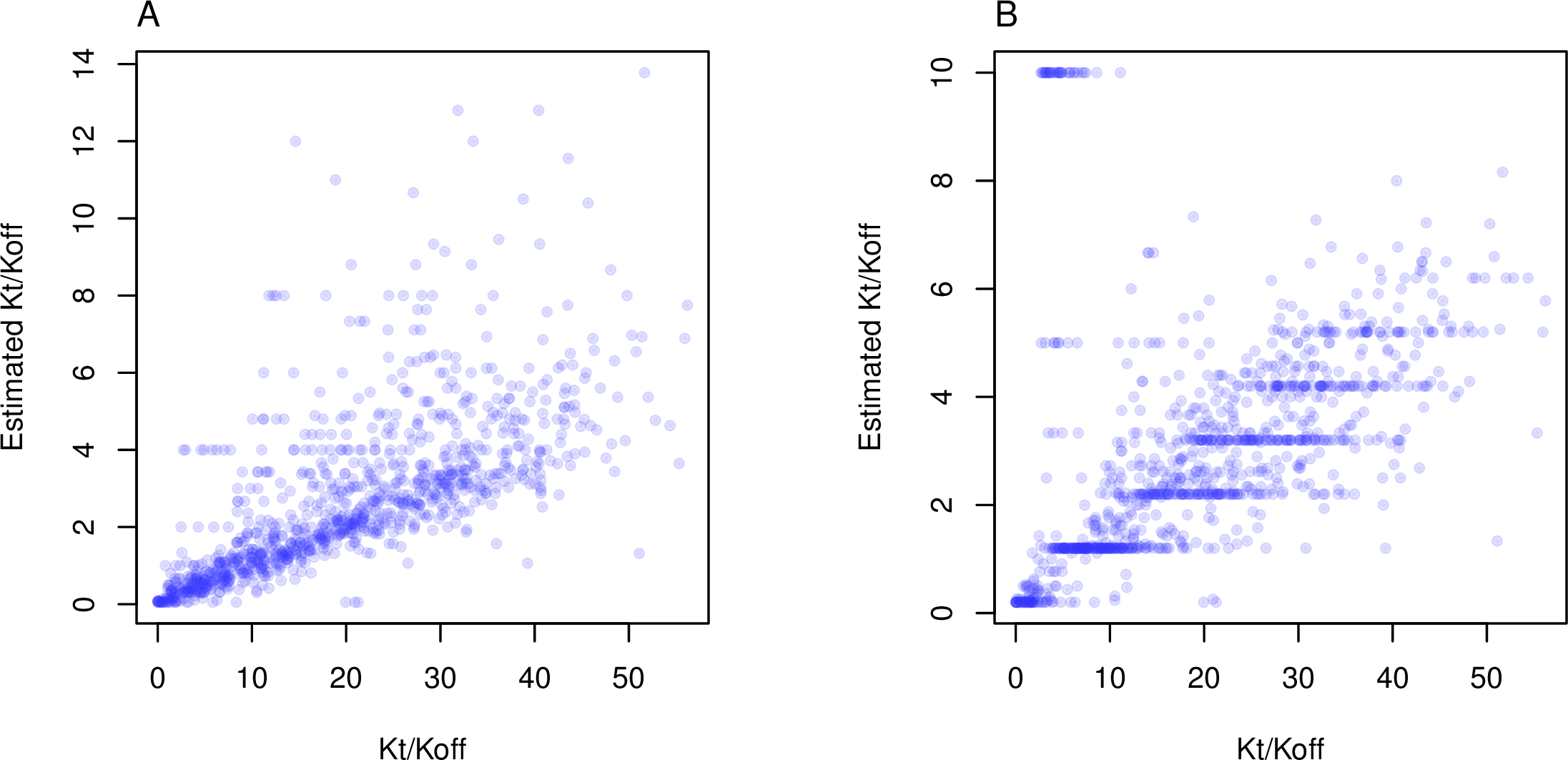

Supplement: S2 Fig — These figures compare known and estimates Kt/Koff ratios for the same datasets depicted in S1 Fig with (A) depicting simulations where 0 < Koff < 10 and (B) depicting simulations where 10 < Koff < 20. In both cases, the known and estimated ratio of Kt/Koff is linearly correlated. (TIF) [file pcbi.1005072.s002.tif]

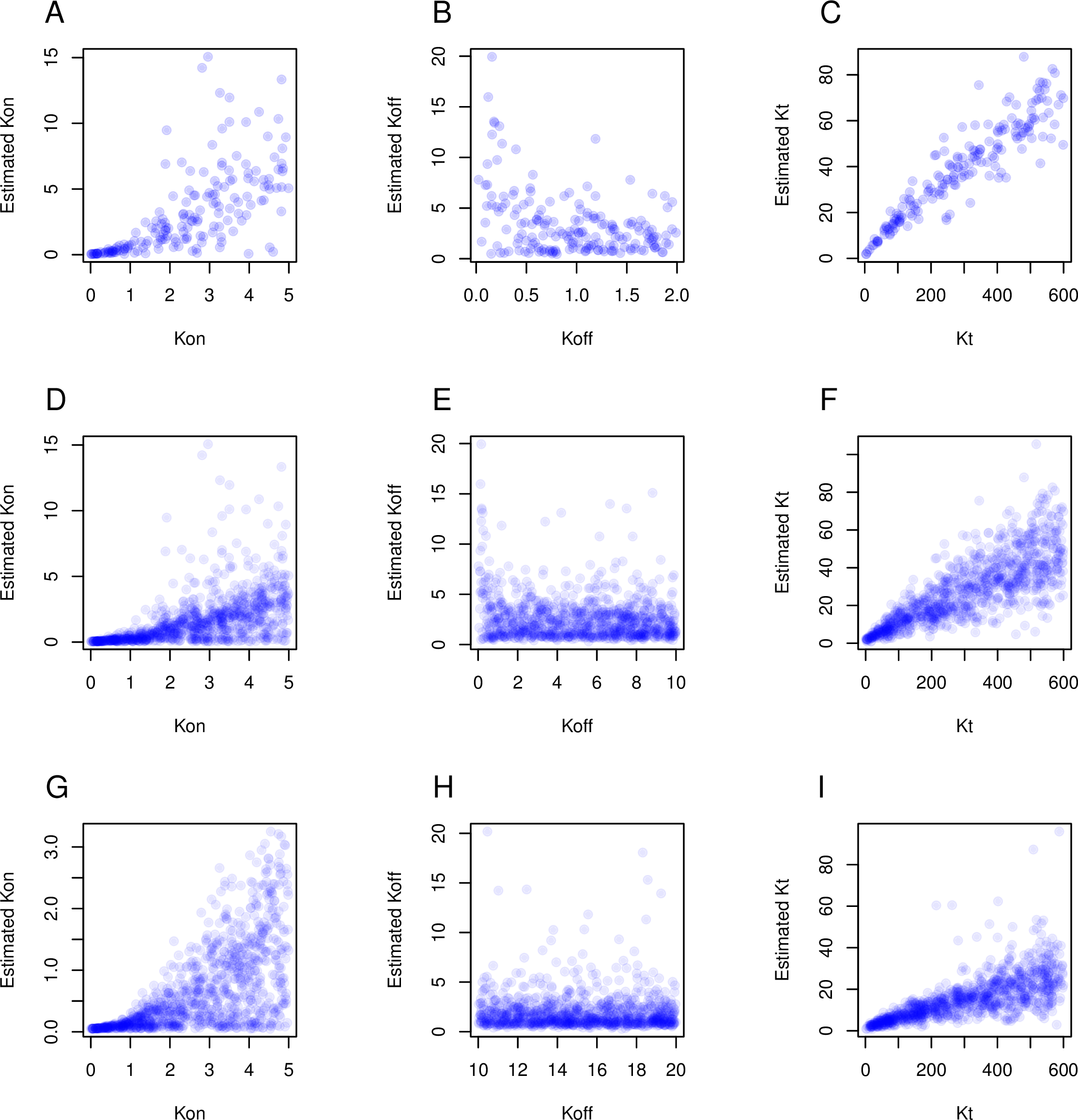

Supplement: S3 Fig — The kinetic parameters of the simulated datasets were estimated using the Gibb’s Sampling approach introduced by [12]. This method was designed for RNA-seq, so the gene length is a required input, but 10,000bp was included for all genes and all the mRNA counts were multiplied by this value. Kon is always between 0 and 5, and Kt is always between 0 and 600, and 90% of the mRNA molecules are randomly removed. Subfigures A-C are for simulations with 0 < Koff < 2, D-F are for 0 < Koff < 10 and G-I are for 10 < Koff < 20. Kon is estimated in subfigures A, D, G, Koff is estimated in subfigures B, E, H and Kt is estimated in subfigures C, F, I. (TIF) [file pcbi.1005072.s003.tif]

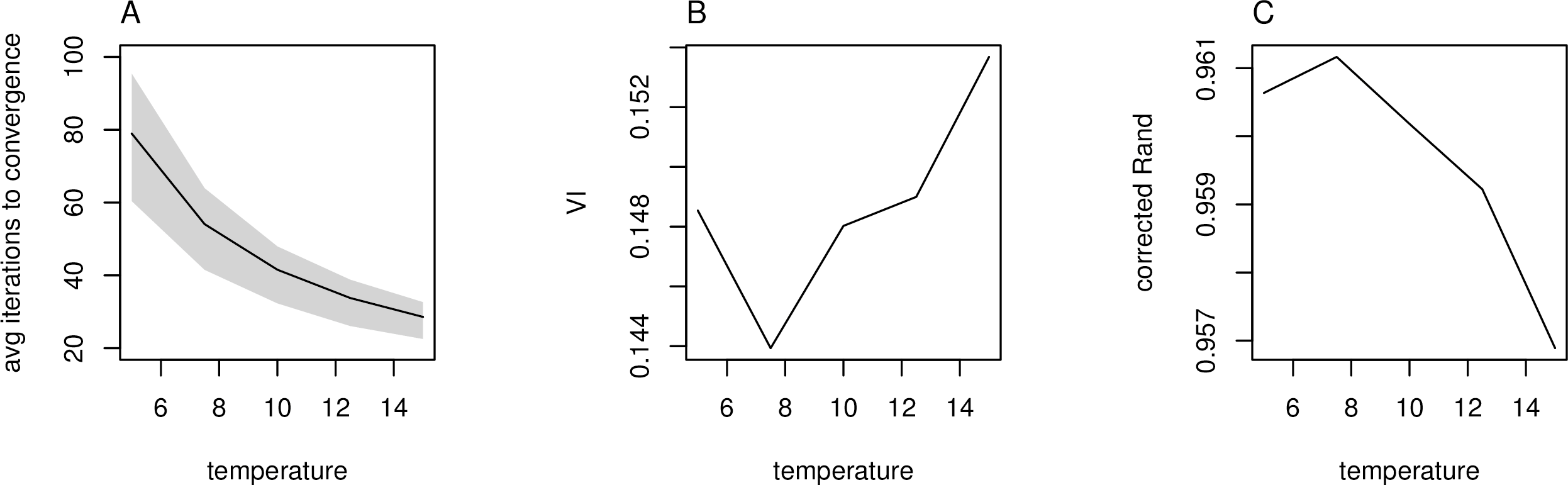

Supplement: S4 Fig — SABEC requires a choice of a temperature parameter (we chose 10 for most of this paper), which speeds convergence of the algorithm. 100 simulation datasets were clustered with SABEC (each repeated 50 times). The y-axes of A shows the average number of iterations before convergence across these 50 repeats. Convergence is defined as the number of iterations of the algorithm until fewer than 5% of the cells swapping clusters, but it is capped at a maximum of 100 iterations. The grey shaded area represents the full range of average values across all 100 simulated datasets and the black line represents the overall average number of iterations. This subfigure illustrates that the larger the temperature, the quicker the algorithm converges. Subfigures B,C illustrate how temperature influences the accuracy of the algorithm, as per the average variable information (VI) and corrected Rand index across all 100 simulated datasets. Both these metrics illustrate that the higher the temperature, the less accurate the algorithm. The aberration when the temperature parameter has a value of 6 comes from the fact that the number of iterations is capped at 100, so in some cases the algorithm did not fully converge. Note that we choose a temperature of 10 elsewhere in this paper. (TIF) [file pcbi.1005072.s004.tif]

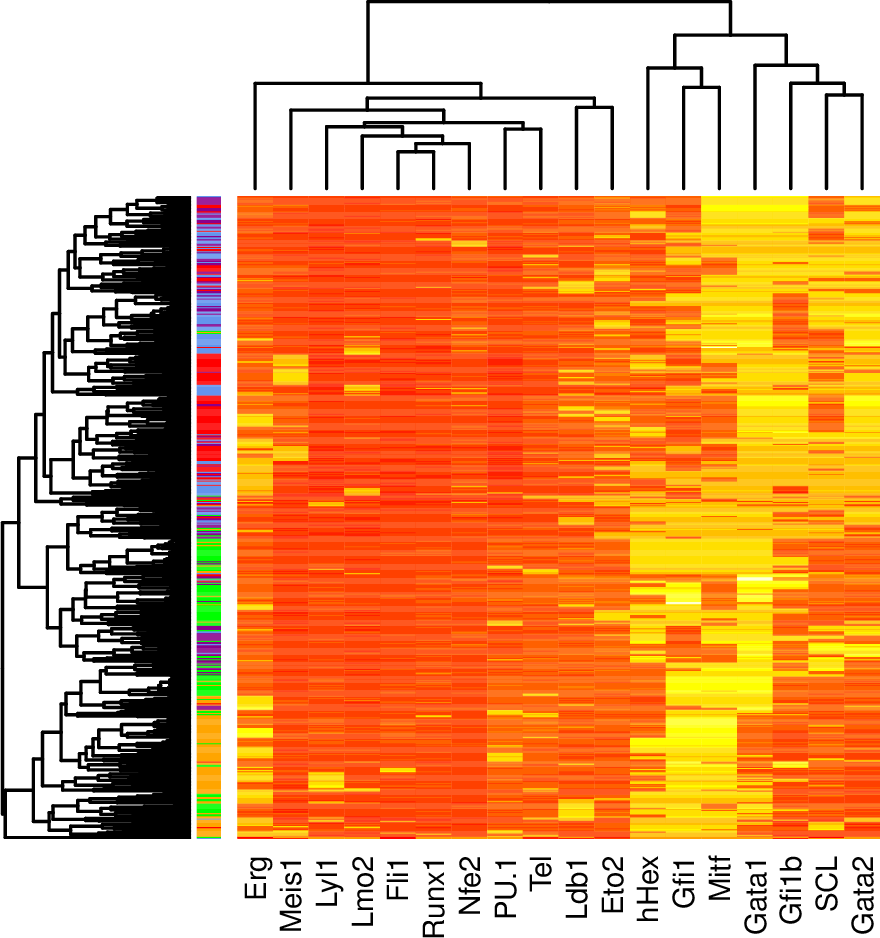

Supplement: S5 Fig — Here are the results of the hierarchical clustering of the normalised qPCR data, color coded the same way as Fig 4. (TIF) [file pcbi.1005072.s005.tif]

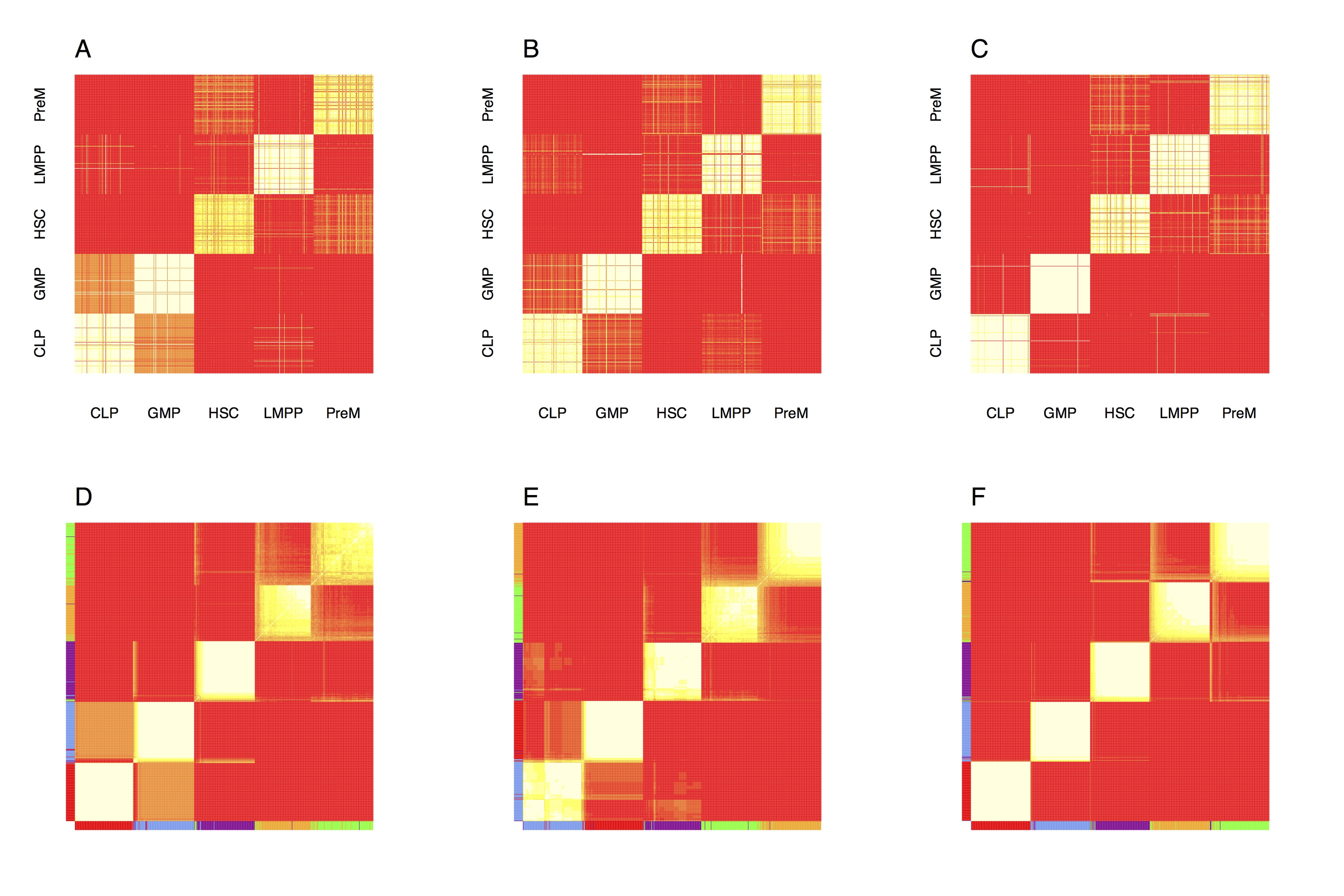

Supplement: S6 Fig — Out of the 100 simulated datasets that were generated, three examples are illustrated here, with their consensus matrices shown in subfigures A-C and the clustered heatmaps of these in D-F. These include the dataset that had the worst robustness by PAC score (A,D), the best robustness (C, F) and a randomly selected third example (B,E). The colours in D-F are blue for CLP, red for GMP, green for HSC, purple for LMPP and orange for PreM. Note that the clustering is more robust than the experimental dataset. In fact, manual inspection of 100 clusterings found no example of HSC being split into two clusters, while GMP/CLP being clustered together (the scenario observed in the experimental dataset), suggesting that the subdivision of HSC into two clusters is probably not an artifact of the SABEC method. (TIF) [file pcbi.1005072.s006.tif]

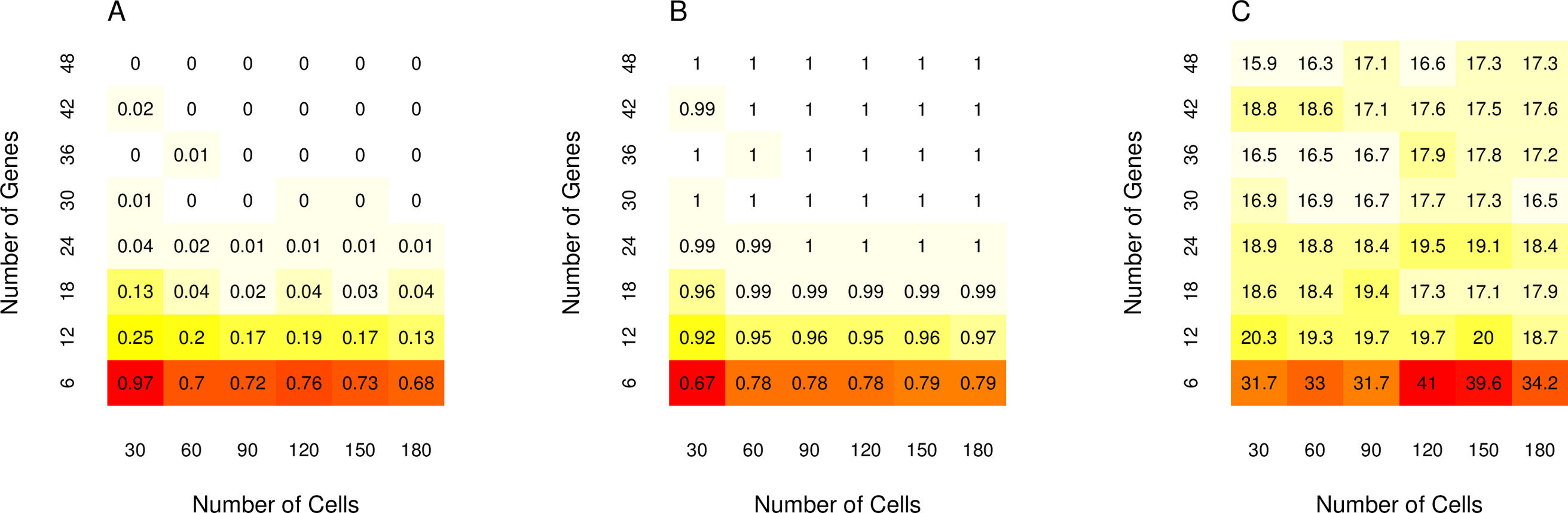

Supplement: S7 Fig — First, we generated a list of 100 parameter sets that were randomly selected from a normal distribution around the experimentally determined kinetic parameter values, with a standard deviation equal to 5% of the parameter range in our look-up table (specifically, 0.25, 1 and 10, for Kon, Koff and Kt respectively). This created a kinetic parameter distribution that was similar to the distribution estimated for the experimental data by [14]. For each simulated dataset, we randomly selected kinetic parameter sets from this list, varying the number of genes and the number of cells, but keeping the number of populations at 5. For each choice of number of genes and number of cells, we repeated this procedure with 5 different simulated datasets. In each case, the SABEC method was used to cluster the dataset (including the consensus clustering step). Subfigure A illustrates the variable information (VI) and B shows the corrected Rand index. Subfigure C shows the average number of iterations of SABEC until convergence. (TIF) [file pcbi.1005072.s007.tif]

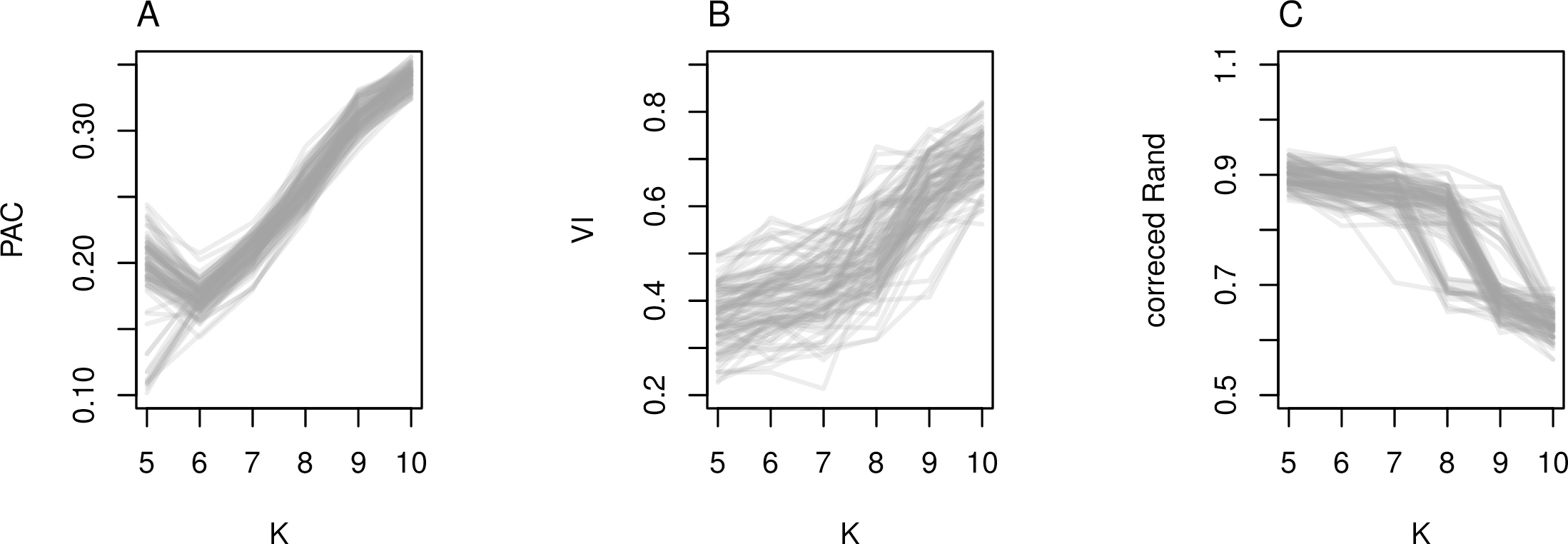

Supplement: S8 Fig — We wished to determine how reliable PAC, VI and the corrected Rand index were at predicting the correct number of clusters when interpreting SABEC data. For instance, it could be that the SABEC method consistently causes there to appear to be more clusters than there actually are, which could mean that the division of HSC into two clusters is spurious. After predicting the number of clusters in the simulated datasets, with each of the 100 resulting curves drawn for PAC (A), VI (B) and the corrected Rand index (C), we see that PAC often predicts a spurious cluster, but VI and the correct Rand index do not. This is one of the reasons we selected K = 6, trusting VI and the corrected Rand index more than the PAC score. (TIF) [file pcbi.1005072.s008.tif]

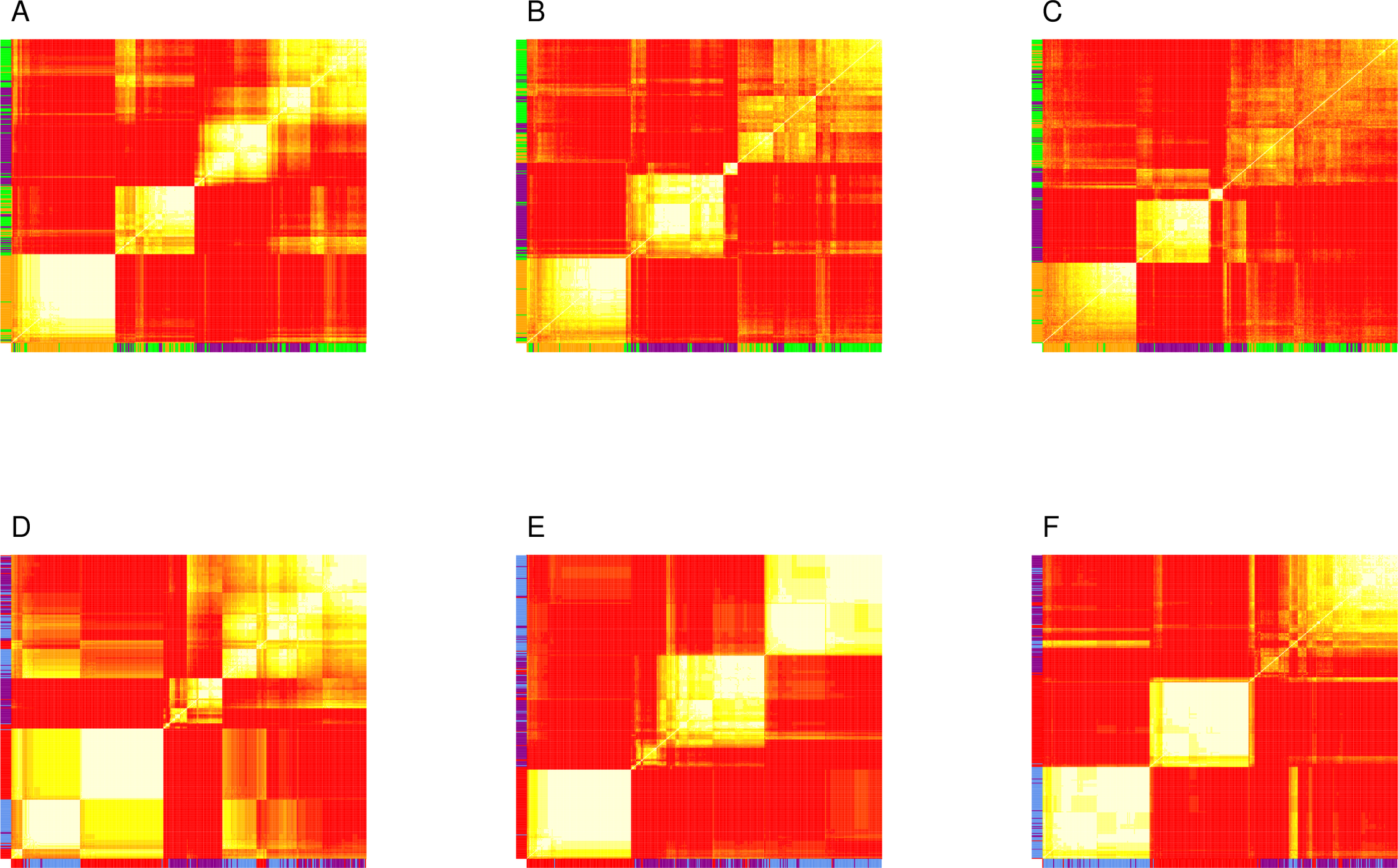

Supplement: S9 Fig — Here we show clustered heatmaps of HSC (green), LMPP (purple) and PreM (orange) cells (A-C) and LMPP (purple), CLP (blue) and GMP (red) (D-F) for the number of clusters K = 5 (A,D), K = 6 (B, E) and K = 7 (C,F). Specifically note how the distinction between the two subpopulations of HSC is defined even in K = 5, but CLP and GMP are not distinguished until K = 6. (TIF) [file pcbi.1005072.s009.tif]

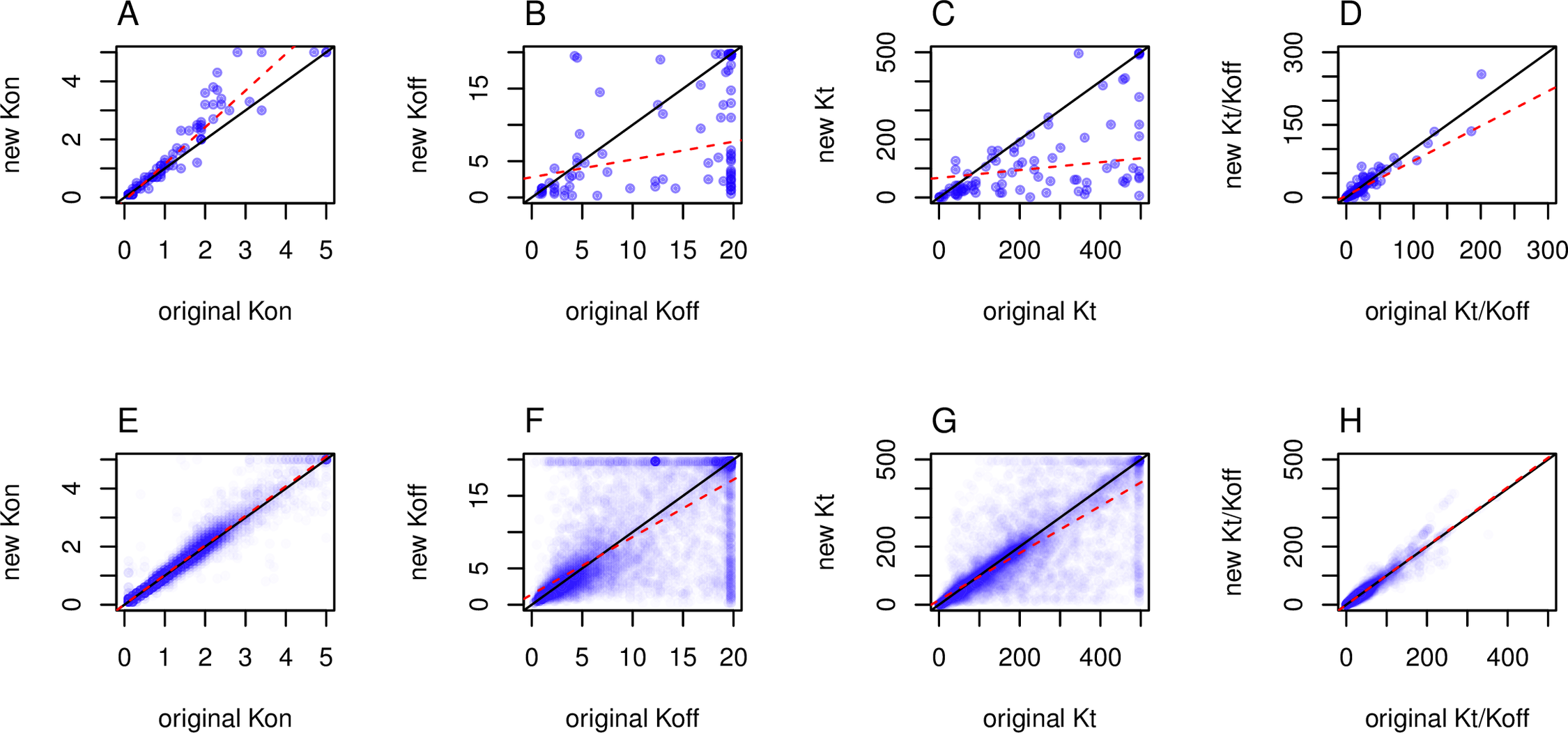

Supplement: S10 Fig — Cells that did not cluster well with other cells of their labelled population were removed in a pruning step, because these cells may not have uniform gene expression bursting kinetics compared to the other cells in their population. However, we were worried whether this would create a consistent bias in the parameter estimates. The line of best fit is designated as the red dashed line. Subfigures A-D compare the original estimates of the kinetic parameters on the complete dataset with the parameter estimates for the pruned datasets in the [14] data, for Kon (A), Koff(B), Kt(C) and Kt/Koff(D). Subfigures E-H also compare the parameter estimate changes in the complete and pruned datasets, but for the 100 simulated datasets. Note that removing outliers does bias the kinetic parameter estimates, in the same direction as observed in the experimental data, but to a much smaller extent. In particular, estimated Kon parameters increase and Koff and Kt values decrease after the pruning procedure, in both cases. (TIF) [file pcbi.1005072.s010.tif]

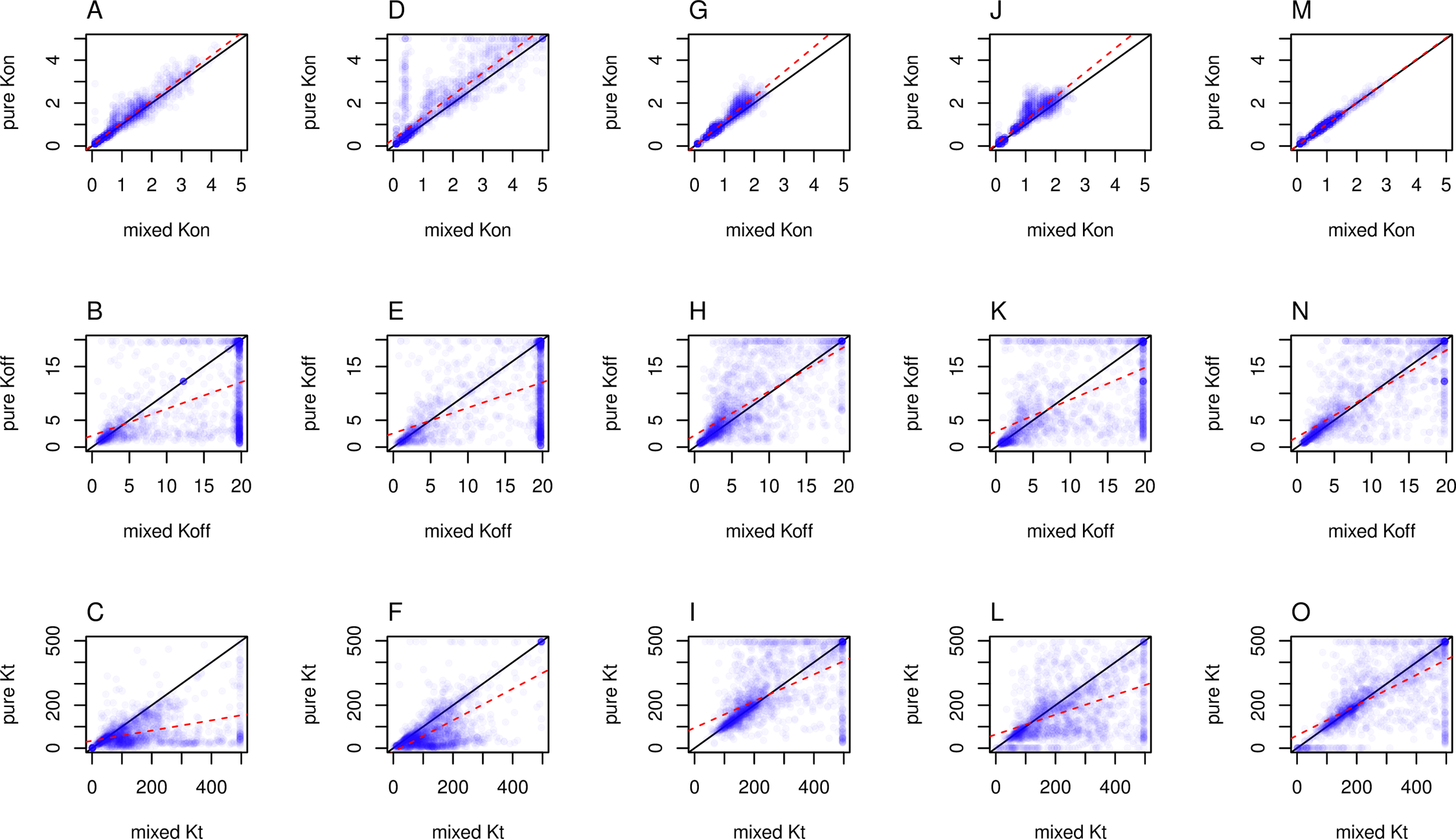

Supplement: S11 Fig — Another potential cause of the change in kinetic parameter estimates after pruning observed in Fig 4 is that the pruning procedure correctly removed cells that did not have homogenous bursting kinetics. In this figure, we compare the kinetic parameter estimates when cell populations are mixed with cells of a different type to the kinetic parameter estimates when the cell populations are pure. For each of the 100 simulated datasets, 10 cells of each population were re-labelled as coming from an incorrect cell type. A comparison of the estimated kinetic parameters in the mixed and pure cell populations are shown for CLP (A–C), GMP (D–F), HSC (G–I), LMPP (J–L) and PreM (M–O). The red line designates the line-of-best fit. Note that the bias observed in S10 Fig is seen prominently here: Kon estimates are higher in mixed cell populations, while Koff and Kt estimates are lower, and the magnitude of the change is much more consistent with that seen in the experimental data. Nevertheless, we cannot neglect the fact that removing false negative cells can also bias the kinetic parameter estimates. (TIF) [file pcbi.1005072.s011.tif]

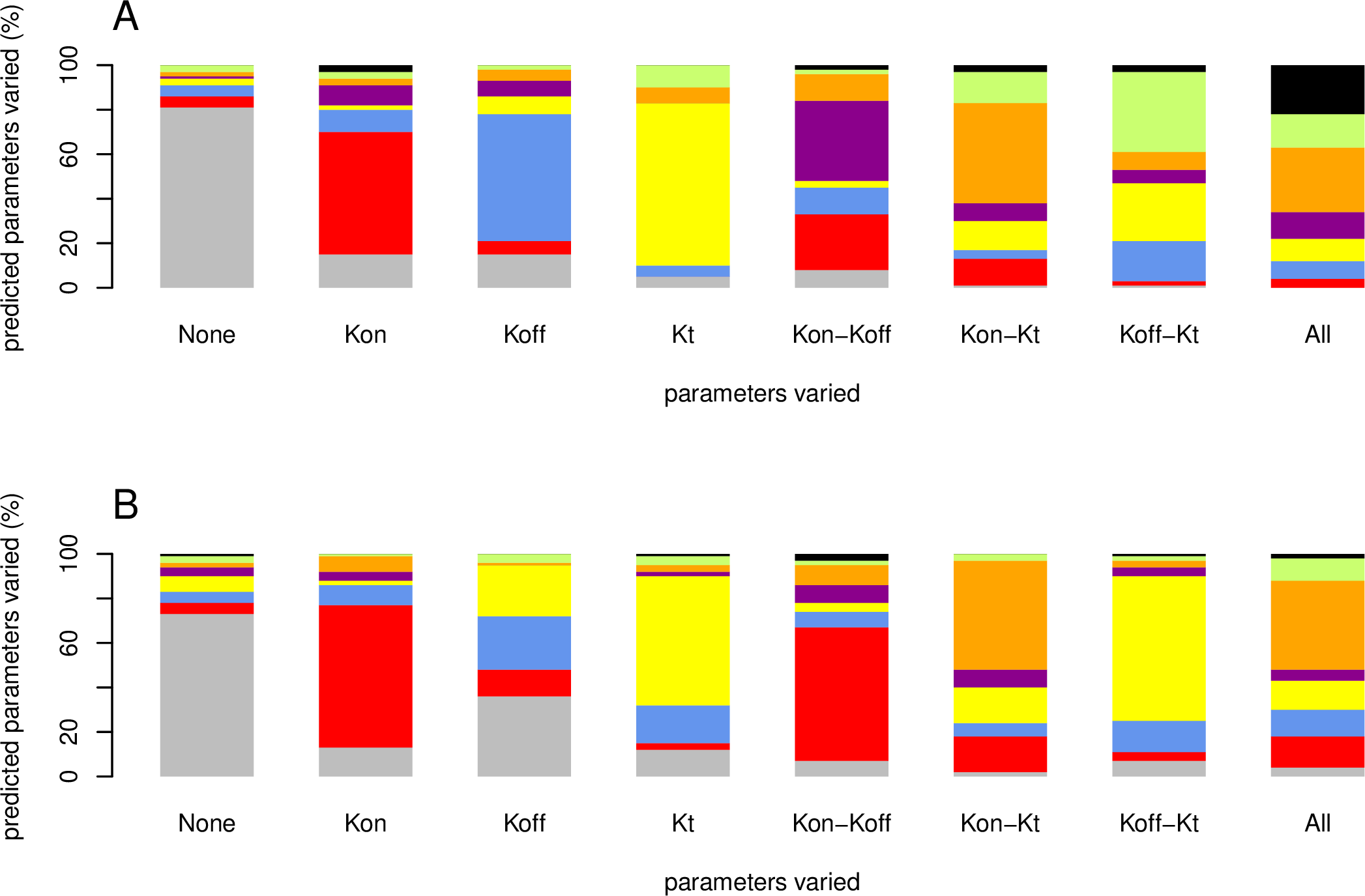

Supplement: S12 Fig — 800 simulated datasets with Koff < 5 (A) and 800 simulated datasets with 5 < Koff < 10 (B) were generated, with 100 examples of each possible type of kinetic parameter set change (x-axis). For each of these, BIC was used to predict the kinetic parameter change, as shown by the colors in the stacked bar plot. Grey represents the case that none of the parameters change and black represents the case that all the kinetic parameters change. The primary colors (red, blue, yellow) correspond with the case of one kinetic parameter changes (Kon, Koff and Kt, respectively). The secondary colors correspond with the cases where two kinetic parameters change, with purple as Kon and Koff, orange as Kon and Kt and green as Koff and Kt. Clearly, BIC enriches for the correct set of parameters, although it is a bit conservative when more than one kinetic parameter is varied. Note that when Koff > 5, Koff is often mislabeled as none and sometimes as Kt. (TIF) [file pcbi.1005072.s012.tif]

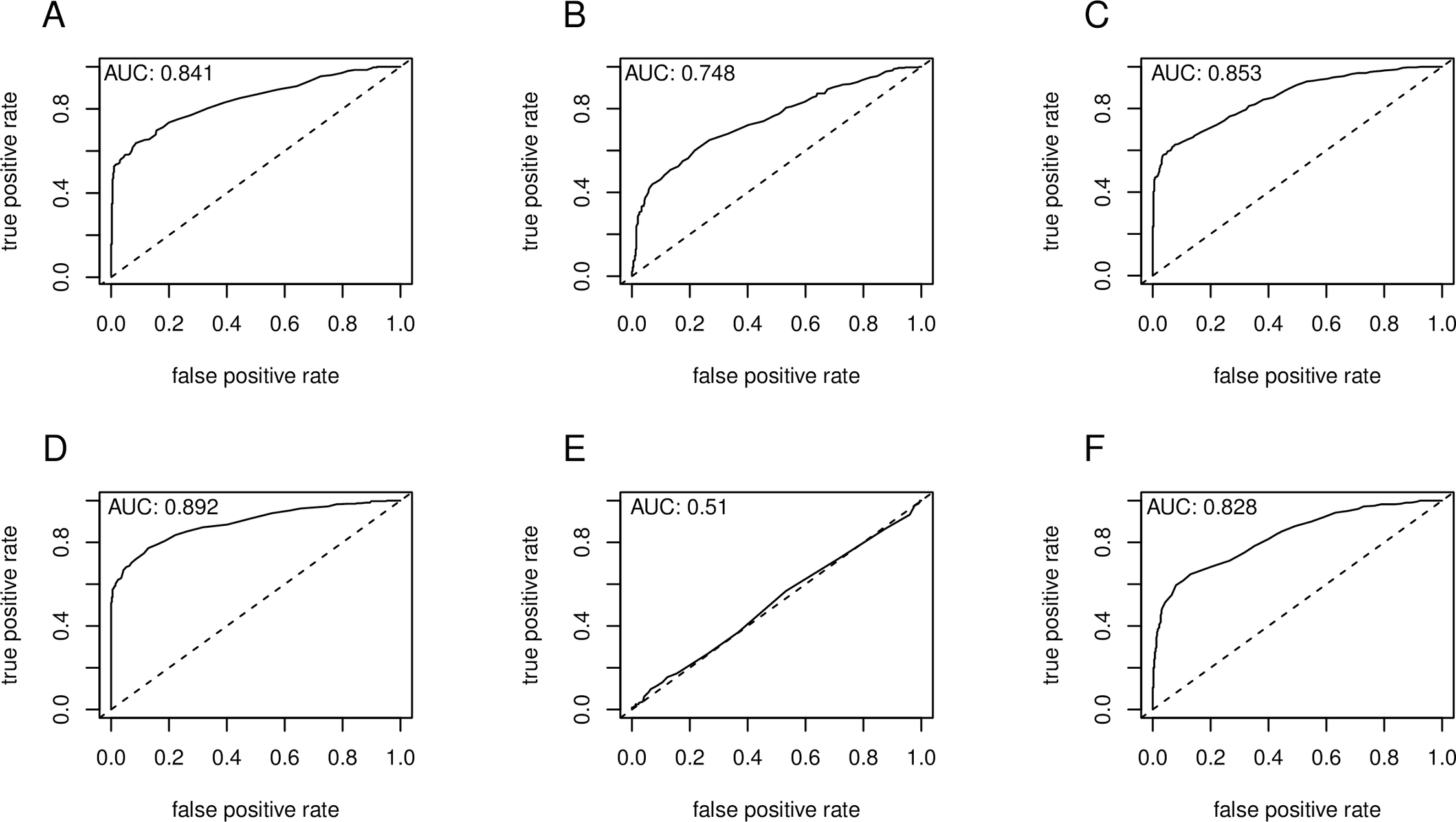

Supplement: S13 Fig — These are the ROC curves for Koff < 5 (A–C) and Koff > 5 (D–F), with the area under the curve (AUC) listed for each figure. Based on the high values of AUC, this method is predictive for Kon and Kt, but less so for Koff. Note that predictive power for Koff completely disappears at higher values, although it does not seem to substantially increase Kt false positive rates. (TIF) [file pcbi.1005072.s013.tif]

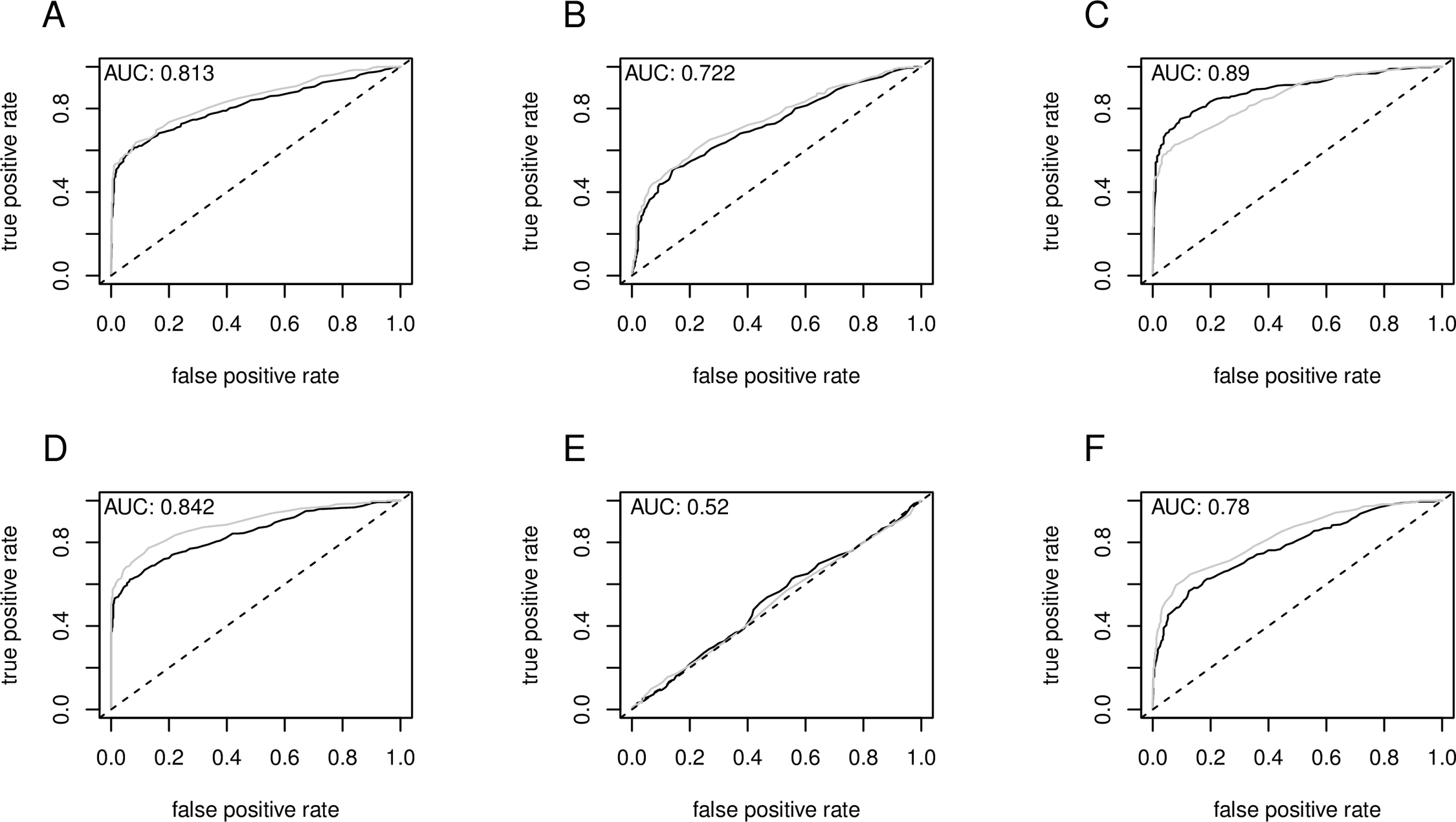

Supplement: S14 Fig — In black are the ROC curves for Koff < 5 (A–C) and Koff > 5 (D–F), with the area under the curve (AUC) listed for each figure. The corresponding MP ROC curves are drawn in grey for comparison. The results are fairly similar, although notably the results are worse than MP for Kt when Koff > 5. (TIF) [file pcbi.1005072.s014.tif]

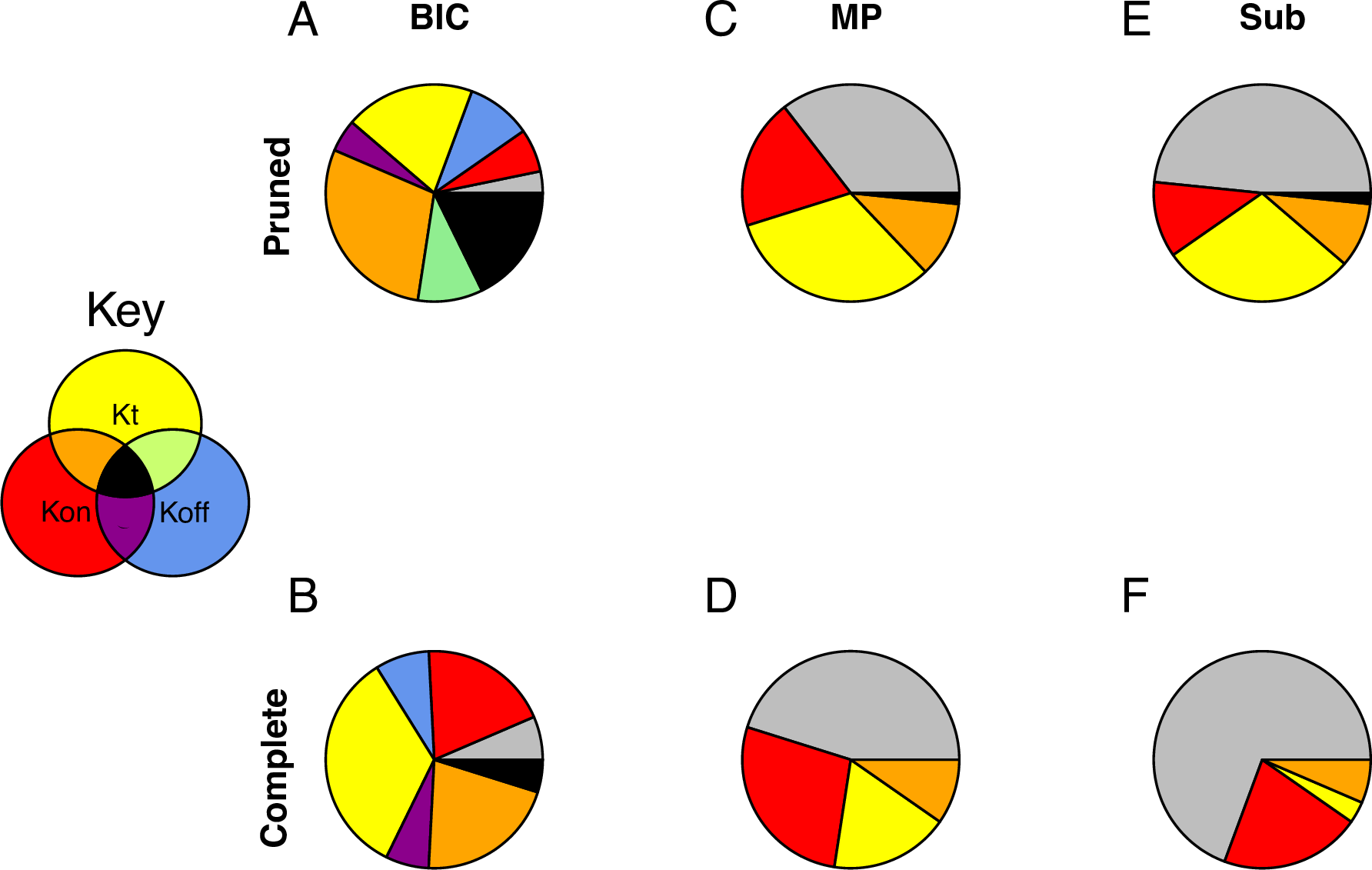

Supplement: S15 Fig — These pie charts display the proportion of different kinetic parameter combinations deemed to have varied for each TF between pairs of populations of cells connected by lines in Fig 4(Bi). The colors designate which kinetic parameter was adjusted, yellow for Kt, red for Kon, blue for Koff, orange for Kt and Kon, green for Kt and Koff, purple for Kon and Koff, grey for no changes and black for all parameters. Subfigures A–B are for BIC, C–D for marginal probability (MP) and E–F for the subset method (Sub). Each of the methods was applied to the dataset where potential outliers were pruned (A, C and E) and the complete datasets before the pruning step (B, D and F). (TIF) [file pcbi.1005072.s015.tif]

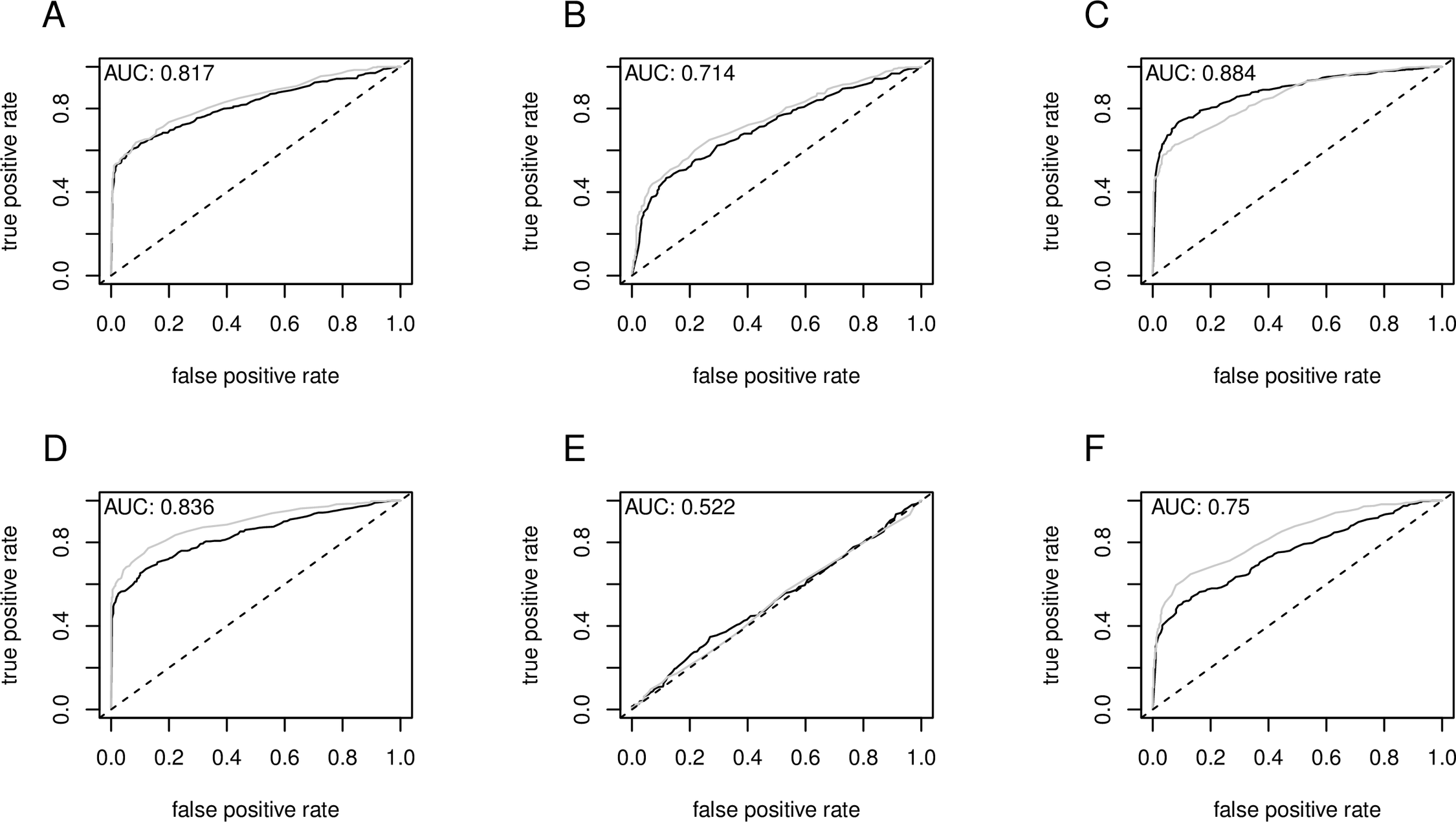

Supplement: S16 Fig — In order to determine whether the specificity and sensitivity of the method depends significantly on the size of the subsamples, we re-ran the subsample method, but sample half the cells (62) instead of a quarter of the cells, as shown in S14 Fig. The grey line still represents the ROC curve for the MP method. The AUC scores are similar across both subset sizes. (TIF) [file pcbi.1005072.s016.tif]

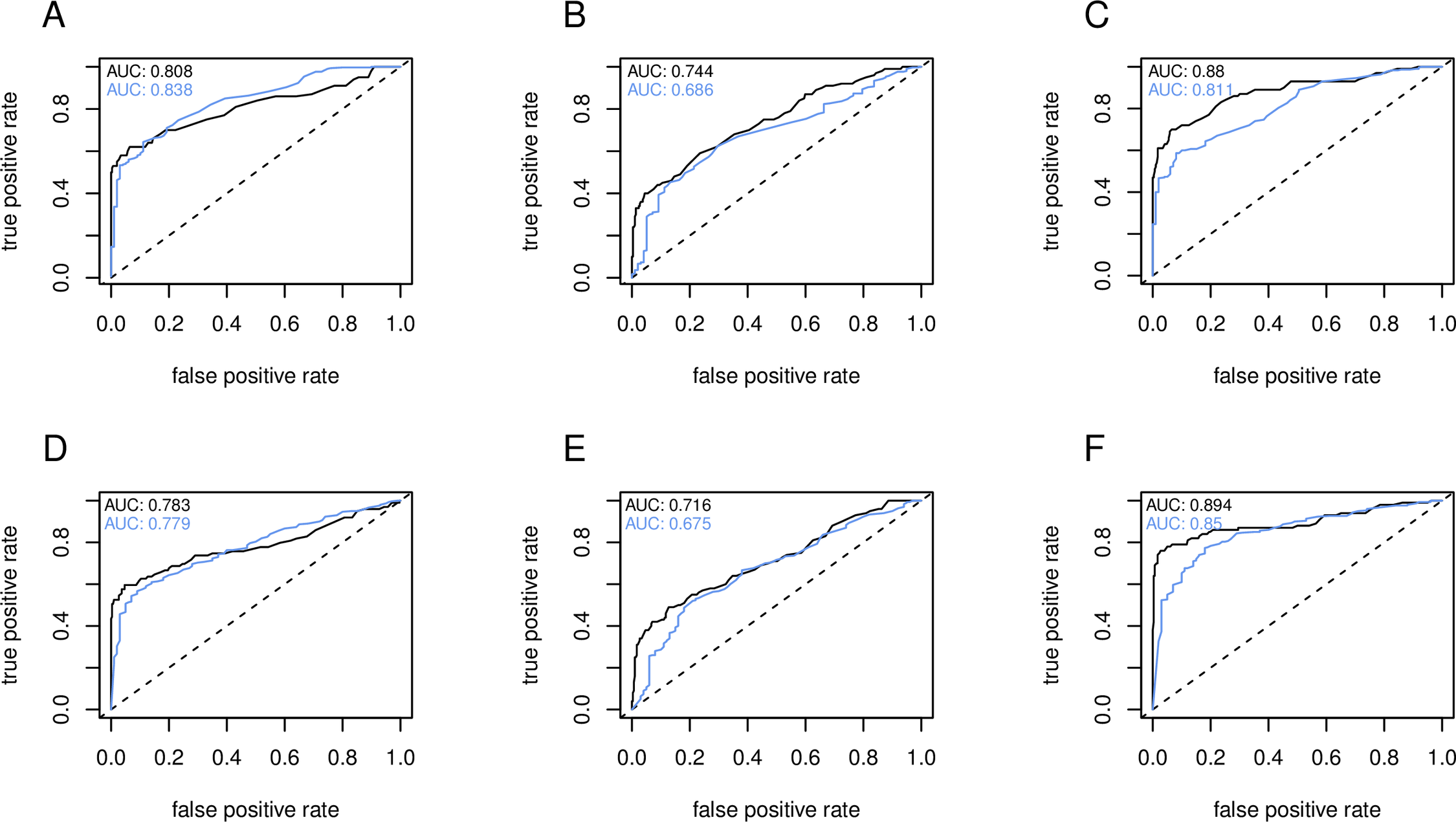

Supplement: S17 Fig — The simulated datasets which were used to test the accuracy of these three methods have an equal number of examples from each of the eight possible sets of parameters that can change. However, we saw in S12 Fig that BIC misses many true positives when more than one kinetic parameter varies. We wondered whether the accuracy of the MP and subset methods are influenced by the assumptions of the number of kinetic parameters that were varied. The black lines and corresponding black AUC values consider the 400 simulated datasets where only 0 or 1 kinetic parameters are varied, while the blue lines and blue AUC values consider the other 400 simulated datasets, where 2 or 3 kinetic parameters are varied. Subfigures A–C show the results for the MP method and D–E show the results for the subset method. In both cases, only the examples when with Koff < 5 are shown. Estimates for Kon appear to be similar under both assumptions, but the estimates for Koff and Kt drop slightly when there are more kinetic parameters that vary (the blue line). (TIF) [file pcbi.1005072.s017.tif]

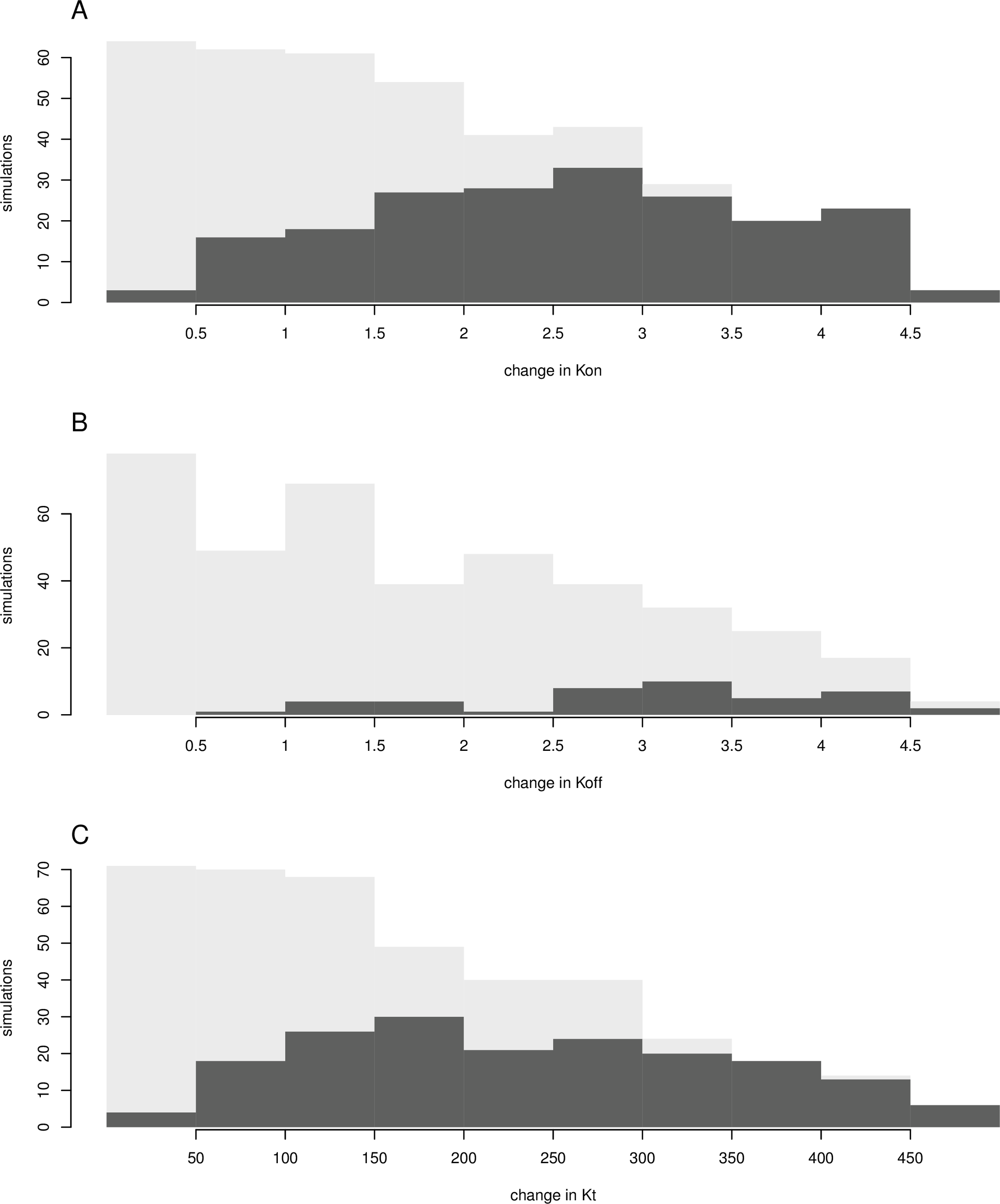

Supplement: S18 Fig — This histogram shows the full distribution of the magnitude of kinetic parameter variation among simulated datasets in which that kinetic parameter varied (positives) for Kon (A), Koff (B) and Kt (C). The samples that were correctly identified by the intersection of the three methods (the true positives) are shaded dark grey, while the false negatives are light grey. Note that the proportion of true positive values increases as the magnitude of the kinetic parameter change increases. (TIF) [file pcbi.1005072.s018.tif]

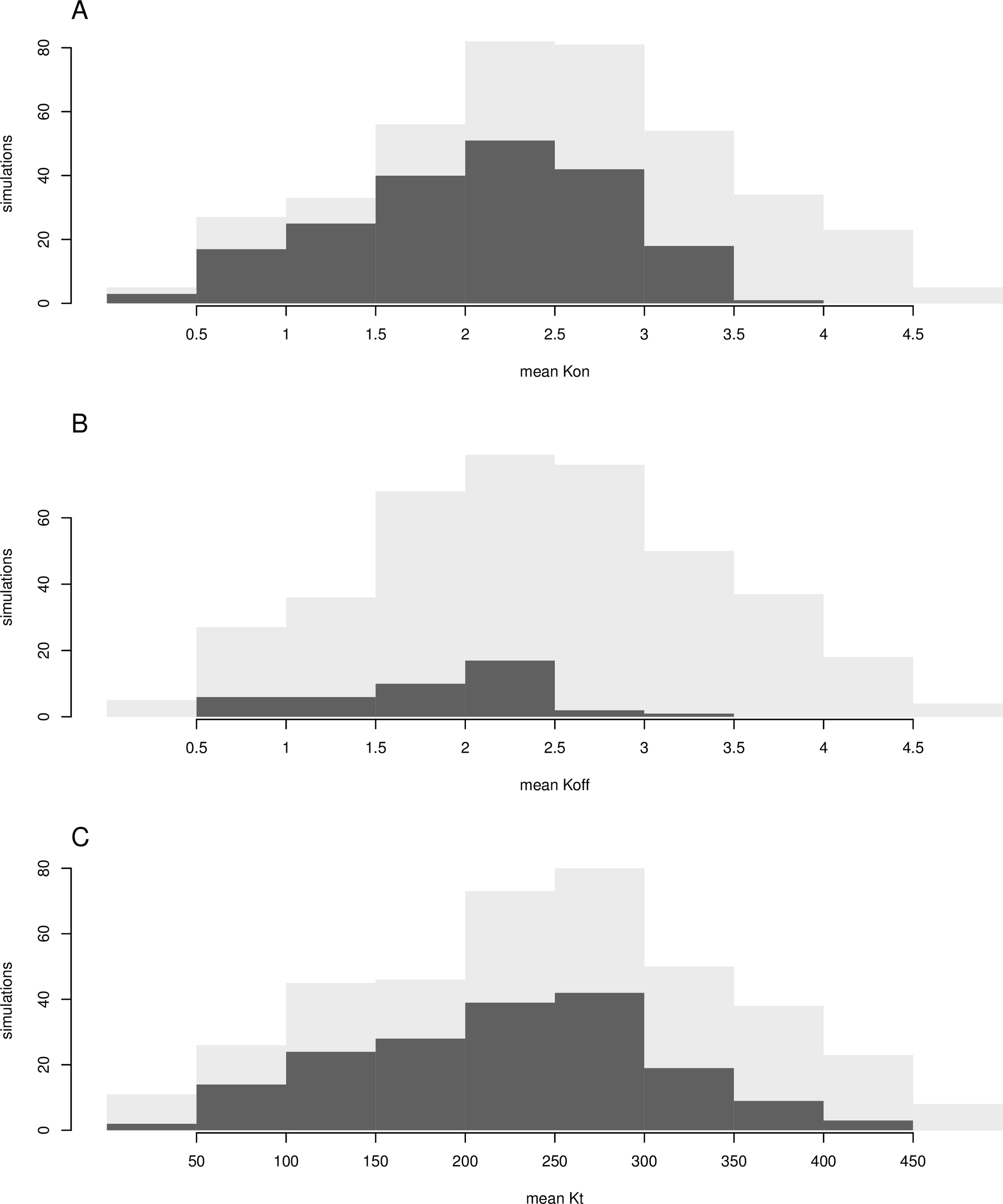

Supplement: S19 Fig — This figure is parallel to S18 Fig, except instead of subtracting the two kinetic parameters assigned to the pair of populations, these two values are averaged. For Kon (A), Koff (B) and to a lesser extent Kt (C), there is a higher rate of detection when the values are closest to the origin, the region where the kinetic parameters are more easily discernible. (TIF) [file pcbi.1005072.s019.tif]

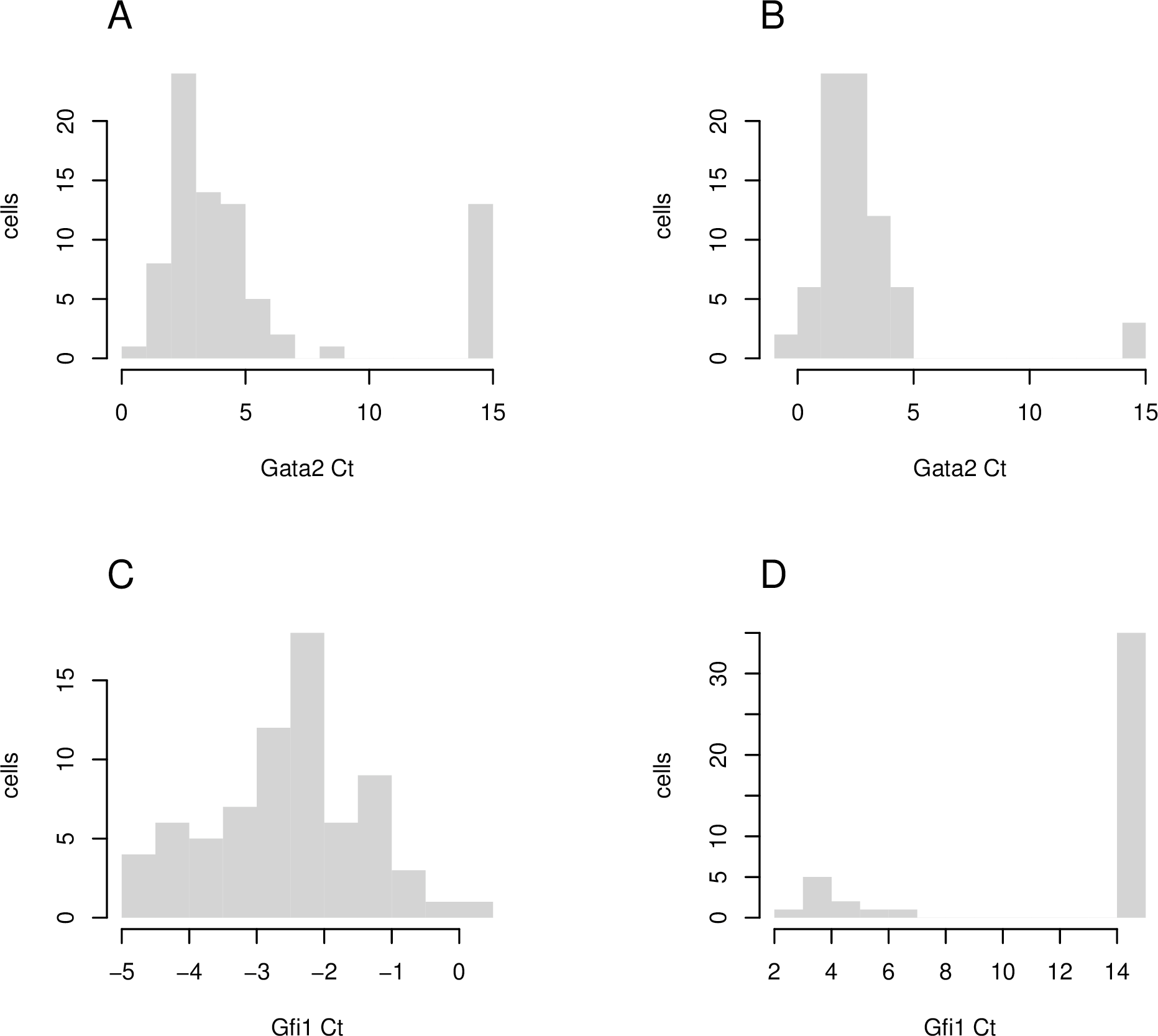

Supplement: S20 Fig — These histograms depict the normalised Ct values for the GATA2 down regulation experiment (A), the GATA2 control HPC7 cells (B), the GFI1 up regulation experiment (C), and the GFI1 control HPC7 cells (D). The values where Ct is 15 are cells where there was no visible expression of the gene. GFI1 up regulation was successful for all of the cell, but GATA2 down regulation only slightly decreased its overall expression compared to the controls. (TIF) [file pcbi.1005072.s020.tif]
